# Structural Characterization of 1,2-Bis(4-cyanophenyl)diazene Oxide and Its Cyclotrimerization to a Triazine-Linked Polymer

**DOI:** 10.1021/acsomega.5c11736

**Published:** 2026-07-29

**Authors:** Mladen Borovina, Vesna Petrović Peroković, Barbara Panić, Ivana Biljan

**Affiliations:** Department of Chemistry, 117036 University of Zagreb Faculty of Science, Horvatovac 102a, Zagreb HR-10000, Croatia

## Abstract

Azoxybenzenes have been widely studied due to their diverse applications, but reports on their detailed structural characterization are rare. It is often the case that even when the structure is obtained, the azoxy group is disordered, which makes it difficult to determine its geometrical parameters correctly and, in some cases, even its chemical identity (due to oxygen disorder at both nitrogen sites). Here, the molecular structure of a nitrile-substituted azoxybenzene derivative, 1,2-bis­(4-cyanophenyl)­diazene oxide (**1**), was accurately determined by single-crystal X-ray diffraction (SCXRD) performed by using crystals of a dichloromethane solvate (**1a**), which displays channels that propagate along the *a*-axis. SCXRD measurements showed no significant changes in the unit cell parameters of **1a**, even at 127 °C. TG measurements reveal that **1a** remains stable up to ∼190 °C, at which point dichloromethane molecules start to leave the structure, and a disordered structure (**1b**) forms. Compound **1** was used for the synthesis of azoxy-linked triazine-based polymer (**2**) by a trifluoromethanesulfonic acid-catalyzed trimerization reaction under microwave conditions. The obtained polymer **2** is an amorphous solid with good thermal stability, which exhibits a rather low BET surface area of 10.2 m^2^ g^–1^ and shows no affinity for CO_2_ adsorption. The preliminary investigation revealed that polymer **2** has a band gap of 2.69 eV, rendering it a wide-bandgap semiconductor.

## Graphical abstract

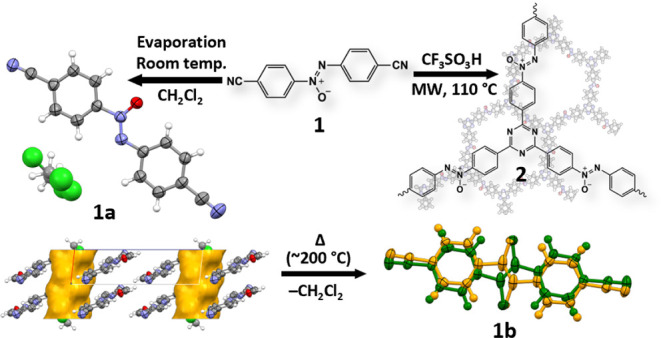

## Introduction

Aromatic azoxy compounds, bearing an unusual 1,3-dipolar O–N=N linkage, are useful intermediates and precursors for the fabrication of dyes, pigments, polymer inhibitors, reducing agents, materials for electronic devices, pharmaceuticals, liquid crystals, chemical stabilizers, and food additives.^1–5^ Furthermore, azoxy functionality can be used in synthetic chemistry as an *ortho*-directing group in the C–H functionalization of arenes^6–9^ and for the synthesis of hydroxy-substituted azobenzenes by the Wallach reaction.^10^ Aromatic azoxy compounds are mainly synthesized by *in situ* condensation of nitrosoarene and *N*-arylhydroxylamine derivatives, which are usually generated by oxidation of anilines or reduction of nitroarenes.^10,11^ In addition, the reductive dimerization reaction of nitrosobenzenes was recently reported as an effective method for the synthesis of azoxybenzenes.^12,13^ Crystallographic data for this class of compounds are, however, somewhat less abundant. CSD searches resulted in only 251 hits (140 without disorder) for structures containing azoxy groups, of which 136 hits (59 without disorder) corresponded to aromatic azoxy compounds.^14^

Previous computational studies suggested that incorporating an azoxy moiety into porous organic polymers (POPs) could improve their efficiency in capturing carbon dioxide (CO_2_), the main greenhouse gas.^15,16^ In general, since CO_2_ possesses a higher quadrupole moment in comparison to nitrogen (N_2_) and most other gases, the introduction of polar functionalities and Lewis basic sites can lead to an increase in the CO_2_ capture capacity and selectivity of POPs. Among the promising candidates for CO_2_ adsorption are azo-bridged POPs, which exhibit high CO_2_ uptake capacity and selectivity.^17,18^ According to computational and experimental studies, this was attributed to favorable Lewis acid–base interactions between CO_2_ and azo groups.^17,19,20^ Another class of promising nitrogen-rich porous materials for CO_2_ capture are triazine-based POPs, whose structural, physicochemical, and functional features (e.g., surface area, porosity, and CO_2_ uptake capacity) are strongly dependent on the type of monomer precursors and the bonding between them.^21–24^ Triazine-based POPs are often synthesized via trimerization reactions of aromatic nitriles under ionothermal conditions^21^ and superacid-catalyzed conditions.^25^ The latter synthesis method, which uses trifluoromethanesulfonic acid as the catalyst under room temperature and microwave-assisted conditions, offers advantages due to lower reaction temperatures and shorter reaction times when compared to ionothermal synthesis. However, it results in triazine-based polymers exhibiting porosities lower than those synthesized by ionothermal polymerization.

In our recent study focused on azodioxy-linked porous polymers,^26^ we synthesized 4-nitrosobenzonitrile by oxidation of 4-aminobenzonitrile with Oxone (potassium triple salt with the chemical formula 2KHSO_5_·KHSO_4_·K_2_SO_4_). During this reaction, a nitrile-substituted azoxybenzene derivative, 1,2-bis­(4-cyanophenyl)­diazene oxide (**1**), was formed as a byproduct, probably by *in situ* condensation of the nitroso and *N-*arylhydroxylamine derivatives (Figure 1).^1,27^ Noteworthy, compound **1** was previously synthesized,^12^ but its structure was not fully elucidated. Herein, we aimed to contribute to the limited detailed structural characterization currently available for azoxybenzenes by determining the molecular structure of 1,2-bis­(4-cyanophenyl)­diazene oxide (**1**). By evaporation of the solvent at room temperature, compound **1** crystallizes as a dichloromethane solvate (**1a**) and crystals of suitable quality for single-crystal X-ray diffraction (SCXRD) analysis were obtained. Structural features of azoxy compound **1** were also characterized by FTIR and NMR spectroscopy, while its thermal stability was determined by thermogravimetric analysis (TGA). In addition, crystals of **1a** were characterized by using powder X-ray diffraction (PXRD). By heating crystals of **1a** in a nitrogen (N_2_) atmosphere for 90 min at 190 °C or 30 min at 210 °C, single crystals of **1b** were obtained which were of sufficient quality for SCXRD measurements and were also characterized using IR and NMR spectroscopy as well as PXRD. Compound **1** was then subjected to a trifluoromethanesulfonic acid-catalyzed trimerization reaction under microwave conditions to afford azoxy-linked triazine-based polymer **2** (Figure 1). To the best of our knowledge, there are no literature reports on the synthesis of POPs in which building units are connected by azoxy linkages. The obtained polymer was thoroughly characterized by FTIR spectroscopy, ^13^C CP/MAS NMR spectroscopy, elemental analysis, PXRD, TGA, N_2_ gas adsorption–desorption experiments, scanning electron microscopy (SEM), and diffuse reflectance spectroscopy. The results were compared to the previously characterized analogous triazine polymers with azo and azodioxy linkages and computationally modeled azoxy-linked triazine porous frameworks.^15,16,26^

**Figure 1:**
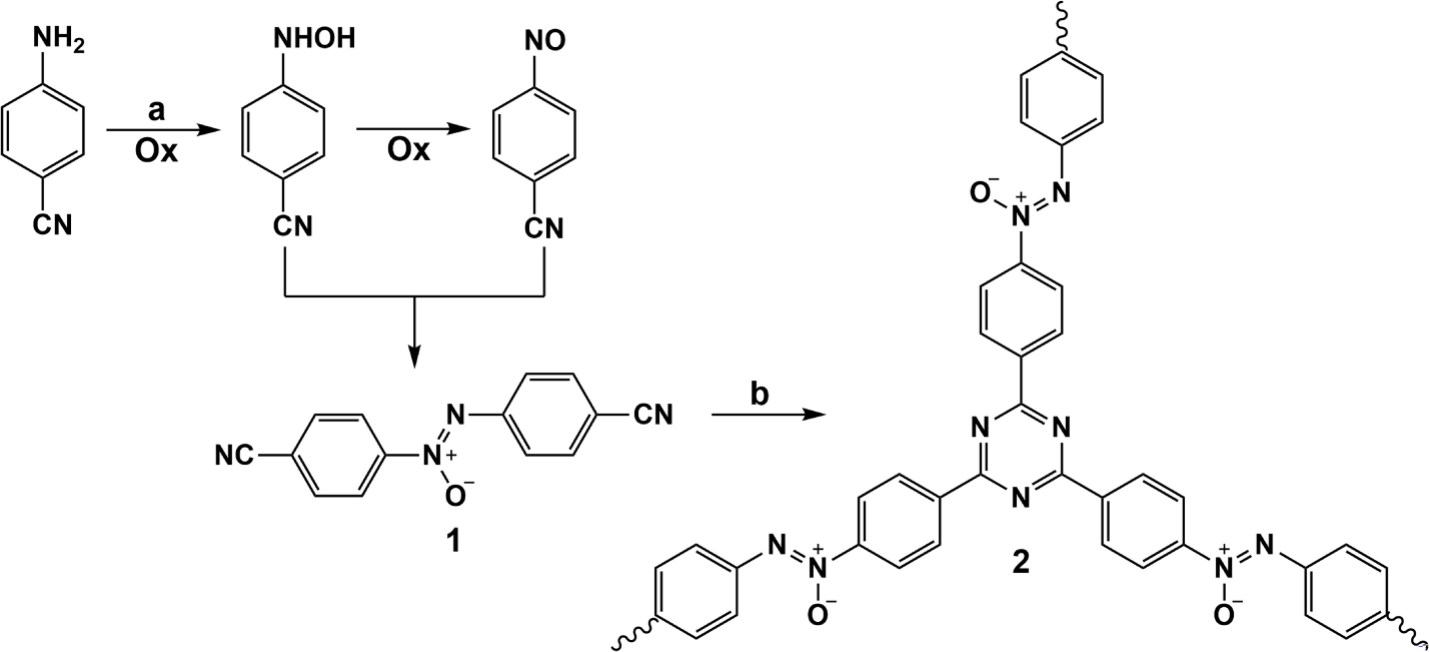
Synthesis route to 1,2-bis­(4-cyanophenyl)­diazene oxide (**1**) and azoxy-linked triazine-based polymer **2**. Reaction a: Oxone, H_2_O, DCM, rt, 20 min. Reaction b: CF_3_SO_3_H, MW, 300 W, 30 min, and 110 °C.

## Experimental Section

### General Information

Compounds **1** and **2** were synthesized by adopting the procedures reported in the literature.^25,26^ Crystals of **1a** were obtained by evaporation from a dichloromethane solution of **1**. Crystals of **1b** were obtained by heating **1a** for 90 min at 190 °C or for 30 min at 210 °C in a nitrogen atmosphere. The details of the synthetic procedures and product data are provided in the Supporting Information. All reagents and solvents for the synthesis of compounds **1** and **2** were used as received from the suppliers. Organic solvents were further purified or dried using standard methods. Microwave-assisted synthesis was performed on a Discover SP microwave reactor equipped with an autosampler, IR temperature sensor, and integrated camera (CEM Corporation). The synthesized compounds were identified by solution ^1^H and ^13^C NMR spectroscopy, solid-state ^13^C CP/MAS NMR spectroscopy, FTIR spectroscopy, and PXRD. Solution-state ^1^H and ^13^C NMR spectra were recorded on a Bruker Ascend 400 MHz NMR spectrometer at 25 °C. CDCl_3_ and DMSO-*d*_6_ were used as solvents, and TMS was used as an internal standard for chemical shifts. Solid-state ^13^C CP/MAS NMR spectra were recorded on a Bruker Avance III HD 400 MHz NMR spectrometer at a spinning rate of 15 kHz. FTIR spectra were recorded on a PerkinElmer UATR Two spectrometer in the spectral range between 4000 cm^–1^ and 400 cm^–1^ at a resolution of 4 cm^–1^, averaging 10 scans per spectrum. Elemental analysis was provided by the Analytical Services Laboratory of the Ruđer Bošković Institute, Zagreb, Croatia.

### SCXRD Analysis

Single crystals of **1a** and **1b** were mounted on a glass fiber and fastened with super glue. Data collection for compound **1a** was carried out at room temperature, 296 K, and for compound **1b** at 170 K, on an XtaLAB Synergy-S Dualflex diffractometer equipped with a PhotonJet (Mo) microfocus X-ray source and a HyPix-6000HE hybrid photon counting (HPC) X-ray area detector. Data collection was performed using the CrysAlisPro system.^28^ The structures were determined using the Olex2 interface (version 1.5).^29^ The starting structural model was obtained using SHELXT^30^ and refined on *F* ^2^ with the SHELXL algorithm.^31^ For further details concerning refinement, see Supporting Information. Mercury, version 2025.1.1,^32^ was used to visualize crystal structures. Thermal ellipsoids were drawn at the 40% probability level. Hydrogen bonds were analyzed using Etter’s Graph-Set notation.^33^ PLATON^34^ was used to calculate geometry parameters of intermolecular contacts. CCDC Nos. 2475649 and 2475650 contain supplementary crystallographic data (which can be obtained at the Cambridge Crystallographic Data Center via https://www.ccdc.cam.ac.uk/structures/).

### PXRD Analysis

Polycrystalline samples were finely ground and placed on a silicon plate for PXRD experiments, which were performed on a Malvern Panalytical Aeris powder diffractometer in the Bragg–Brentano geometry with a PIXcel^1D^ detector under an applied voltage of 40 kV and a current of 15.0 mA. The radiation used was Cu Kα (*λ* = 1.5406 Å), and all patterns were collected at room temperature, from 5° to 40° (2θ), and with a step size of 0.02°. Diffract-WD^35^ was used to process the data and create images.

### Thermogravimetric Analysis

Thermogravimetric analysis was carried out using a simultaneous TGA-DTA analyzer, Mettler-Toledo TGA/DSC 3+. Samples were placed in alumina pans (70 μL) and heated in flowing nitrogen (50 mL min^–1^). Sample **1a** was heated from 25 to 800 °C at a rate of 10 °C min^–1^. Additional experiments were carried out with samples of compound **1a**. One sample was heated from 25 to 190 °C and held under isothermal conditions at 190 °C for 90 min, and another was heated from 30 to 210 °C and held under isothermal conditions at 210 °C for 30 min. In both cases, the heating rate was 10 °C min^–1^. Sample **2** was heated from 30 to 180 °C at a rate of 10 °C min^–1^ and held under isothermal conditions for 10 min at 180 °C to remove traces of solvents. Afterward, it was cooled to room temperature and heated in flowing N_2_ (50 mL min^–1^) from 25 to 800 °C at a rate of 10 °C min^–1^ and held under isothermal conditions for 15 min at 800 °C. CO_2_ sorption experiments were carried out by following a previously reported procedure with minor modifications in experimental conditions.^36^ Before performing CO_2_ adsorption experiments, a 70 μL alumina pan was filled with a fresh sample, heated to 100 °C at a heating rate of 20 °C min^–1^ in N_2_ atmosphere (flow rate 150 mL min^–1^) and held at 100 °C for 30 min to dry the sample. After drying, the CO_2_ adsorption was measured by switching between a N_2_ atmosphere and a CO_2_ atmosphere in 20 min intervals (flow rates for both gases were 150 mL min^–1^) at ∼30 °C. The measured sample temperature varied around 33 °C during the CO_2_ cycles. To correct for different buoyancy effects on the TGA scale and alumina pan, a baseline curve was recorded under the same experimental conditions using an empty alumina pan and subtracted from the measured curve. Data collection and analysis were performed using the program package STARe Software 16.40 Mettler-Toledo GmbH.

### Porosity Measurements

The specific surface area was determined from N_2_ gas adsorption–desorption data obtained with a Micromeritics ASAP-2000 at 77 K. Before analysis, samples were degassed at 150 °C under a dynamic vacuum of 7 mPa. The adsorption data were used to calculate the surface area with the Brunauer–Emmett–Teller (BET) model, using data points within the linear region of the BET isotherm.

### Scanning Electron Microscopy

For insight into the morphology of polymer **2**, a thermal field-emission scanning electron microscope (FE-SEM, model JSM-7000F, JEOL Ltd., Tokyo, Japan) was used. SEM analysis was performed by using an accelerating voltage of 10 kV and a working distance of 10 mm.

### Diffuse Reflectance Spectroscopy

The diffuse reflectance spectrum of a powder sample of polymer **2** was obtained using the UV-3600 (Shimadzu) UV–vis-NIR spectrophotometer with ISR-3100 integrating sphere attachment and barium sulfate as a standard. The band gap of polymer **2** was calculated using the Kubelka–Munk plot.^37,38^

## Results and Discussion

### Structural and Thermal Characterization of Nitrile-Substituted Azoxybenzene Derivative (**1**)

Nitrile-substituted azoxybenzene derivative **1** crystallizes as a dichloromethane solvate (**1a**) in the *P*1̅ space group, with the whole molecule found in the asymmetric unit (Figure 2) and showing no signs of disorder. The crystal structure displays channels that run along the *a*-axis. Dichloromethane (DCM) molecules are disordered in two crystallographically independent positions around inversion centers located in the channels; their occupancies are 0.185(4) and 0.157(4), for a total of 0.34 DCM molecules per formula unit of **1**. This correlates with the data obtained using the PLATON SQUEEZE algorithm,^39^ which estimated the volume of the voids at 98 Å^3^ and the total electron density found in the voids at 28.6 electrons per unit cell, which corresponds to 0.595 DCM molecules per unit cell or roughly 0.3 DCM molecules per formula unit of **1**. By using the Mogul Geometry Check,^40,41^ it was determined that bond lengths and angles in the azoxy groups and the rest of the molecule (Table S2) do not deviate significantly from values found in other deposited structures in the CSD. Molecules of **1** form π-stacking interactions along the *a*-axis; they further associate via C–H···O and C–H···N interactions into two anti-parallel 1D supramolecular chains that form supramolecular ribbons along the *b*-axis. The ribbons are further connected via C–H···N interactions along the *c*-axis, and voids in the structure form between them (Figures 2b and c). Geometrical details of intermolecular interactions between molecules of **1** can be found in the Supporting Information (Table S4). While DCM molecules were difficult to model due to their significant disorder, all chlorine atoms lie in the same plane in orientations that could lead to the formation of potential C–H···Cl and C–Cl···N interactions with molecules of **1**, while hydrogen atoms appear above and below that plane and do not point toward any potential hydrogen bond acceptors. One should also be cautious when considering intermolecular interactions that could potentially form between DCM and the molecules of **1**. However, it appears that DCM could form different interactions depending on the site it fills. Molecules found in the first position (occupancy 0.185(4), labeled A) seem to favor C–H···Cl interactions with Cl2A as the acceptor, while molecules found in the second position (occupancy 0.157(4), labeled B) could form both C–H···Cl interactions and possible C–Cl···N interactions (Figure 2d). No evidence of C–H···N hydrogen bonds with nitrile groups acting as acceptors was found. Calculated PXRD pattern from **1a** is in good agreement with experimental PXRD data (Figure S4). To determine the stability of the crystals, high-temperature SCXRD measurements were made at different temperatures, and unit cell parameters were determined at each point. It was shown that crystals retained their structure up to 127 °C (limit of the instrument), and an increase in cell parameters *a*, *c,* and γ as well as the unit cell volume was observed, while parameters *b*, α, and β decreased (Table S3 and Figure S3).

**Figure 2:**
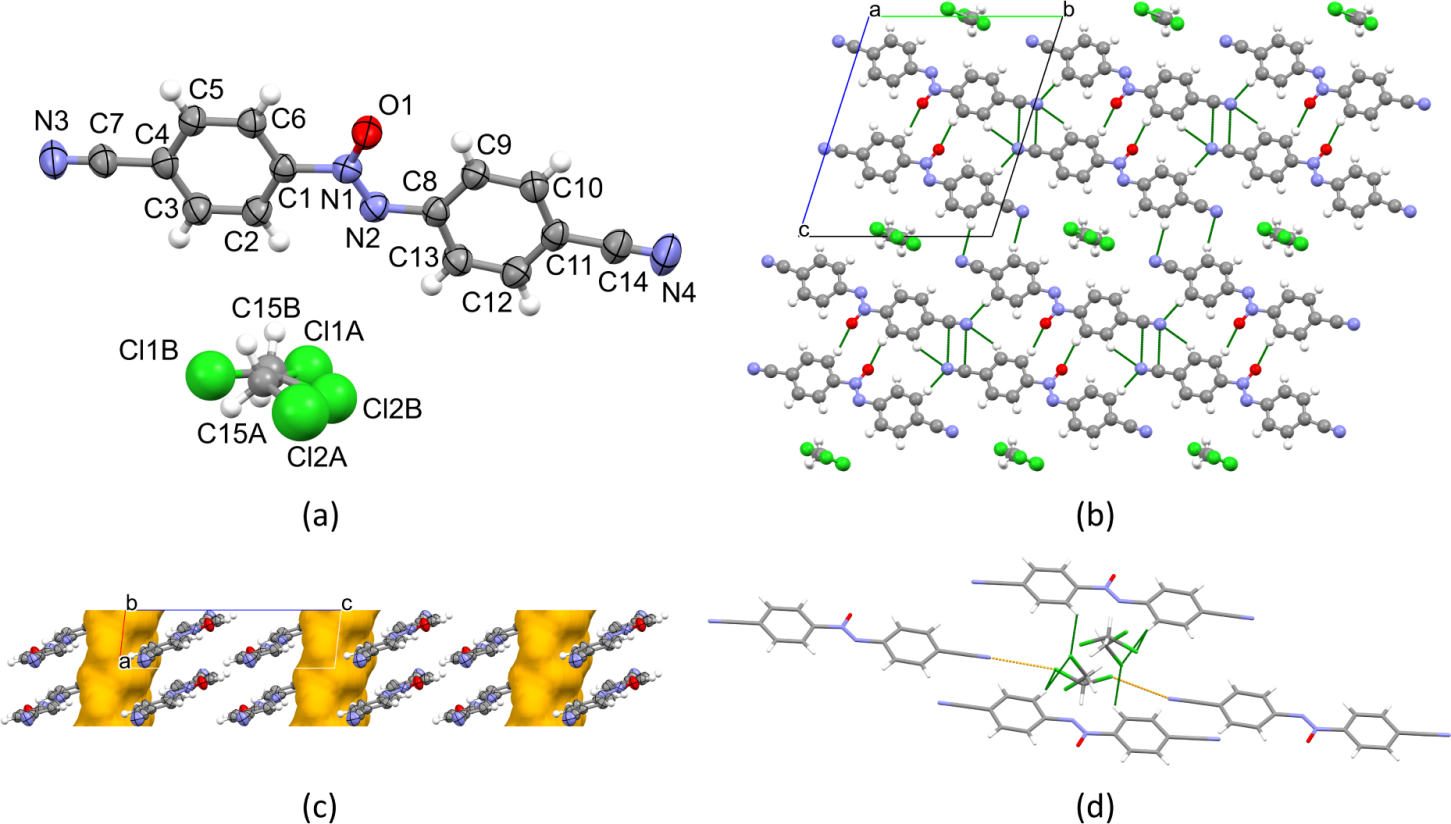
(a) ORTEP-style plot of **1a** with labeling scheme, displacement ellipsoids drawn at 40% probability level at 296 K. Disordered dichloromethane (DCM) molecules were refined using isotropic displacement parameters. (b) Crystal packing viewed along the *a*-axis. DCM molecules are disordered in the channels that run along the *a*-axis. (c) Crystal packing along the *b*-axis. Channels are visualized with orange contours. (d) Interactions between DCM and the molecules of **1**.

When **1a** was heated during TGA experiments in a N_2_ atmosphere, a small decrease in mass was observed at around 190 °C, and the sample lost 3.8% by 220 °C, at which point a sudden increase in the decomposition rate was observed, leading to the complete mass loss by 300 °C (Figure S6). To better study the stability of the compound and determine the nature of the thermal event at 190 °C, experiments were conducted by heating the samples to 190 and 210 °C and holding them under isothermal conditions at those temperatures . When the sample was heated to 190 °C, a sharp decrease in weight of 1.8% was observed, followed by a gradual decrease during the 90 min isothermal step, which resulted in an additional 1.6% decrease in weight (Figure S7). When the sample was heated to 210 °C, it showed a similar loss in weight starting around 190 °C and resulting in a loss of 1.8% by 210 °C. During the isothermal step at 210 °C, the rate of decomposition increased, and the sample lost an additional 3.9% of its weight in 6 min. At this point, the rate of decomposition decreased, but the sample continued to gradually lose weight during the whole step, resulting in a 16% decrease in weight (Figure S8). The additional loss in mass at 210 °C could be attributed to the potential sublimation of the compound.

After the end of the isothermal steps and cooling, the samples were removed and analyzed, and it was determined that they were crystalline. However, a new diffraction pattern was observed (Figure S5). Single crystals of sufficient quality for crystal structure determination were successfully harvested, and their structure was determined (for details, see Table S1). The new form (**1b**) shows two molecules disordered around the inversion center. The occupancies were freely refined and resulted in 53% for the site labeled as A (marked orange in Figure 3) and 47% for the site labeled as B (marked green in Figure 3). The oxygen atoms are further disordered at two additional sites and have halved occupancies, which result in elongated N–O bond distances even when using *dfix* instructions. All other atoms were treated with the *same* instructions, and the aromatic rings and nitrile groups were further treated with the *flat* instructions to obtain a reasonable model of the structure. The crystal packing is mostly determined by π-stacking interactions, which extend along the *a*-axis as in **1a**. The molecules are further connected with C–H···N interactions, which differ significantly between molecules found in different sites, with those in site A exhibiting $R_{2}^{2}\left(8\right)$ motifs which lead to the formation of antiparallel chains, and those in site B showing $C_{2}^{1}\left(6\right)$ motifs with bifurcated hydrogen bonds and herringbone-type packing. It is interesting to note that both sites exhibit similar C–H···O hydrogen bonds, which connect antiparallel chains in A and play a role in stabilizing the packing arrangement in B. To exclude the possibility of a superstructure, diffraction maxima were more closely analyzed, and no additional reflections were observed that would indicate the existence of a superstructure.

**Figure 3:**
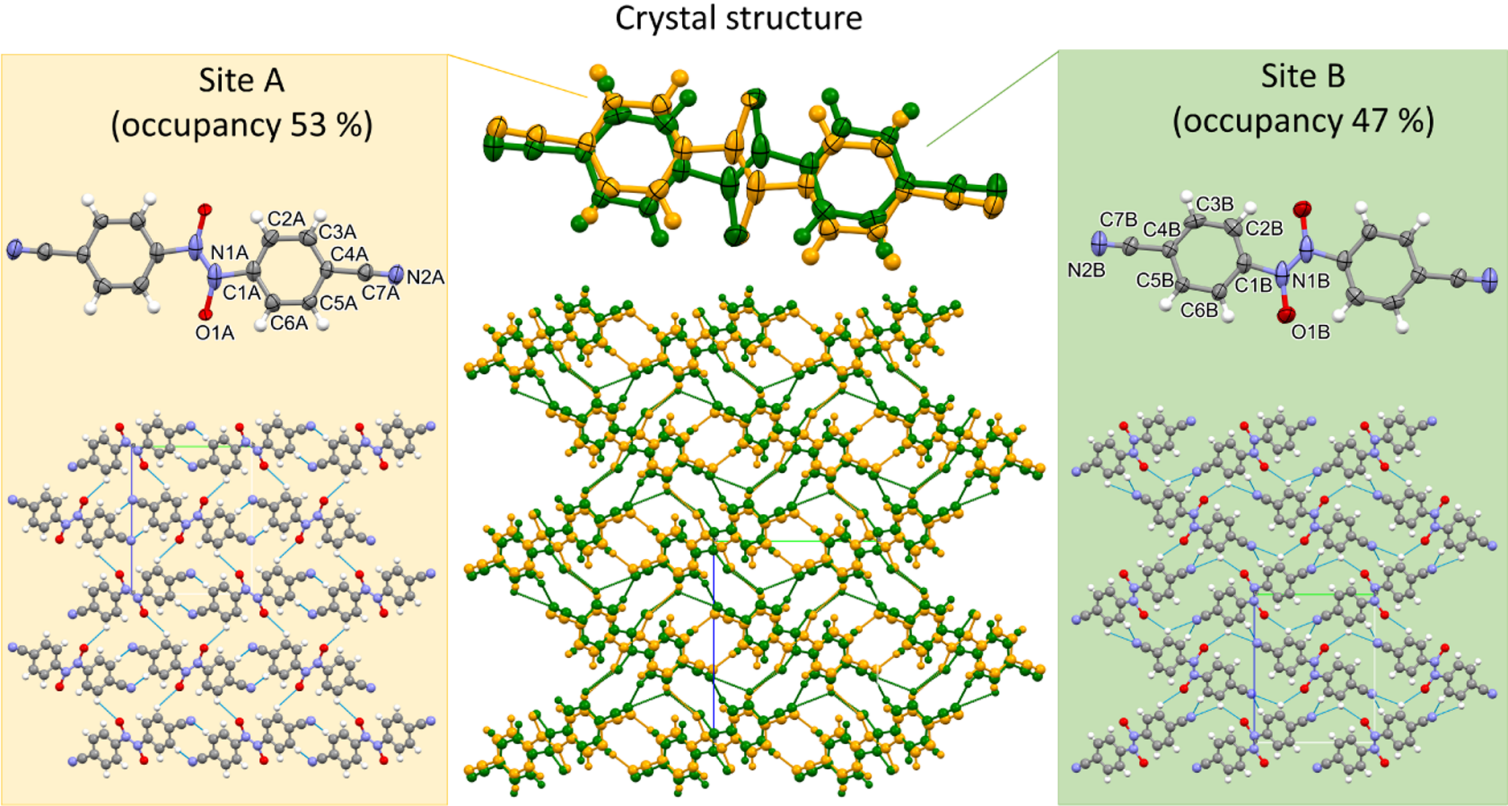
ORTEP-style plot of **1b** with labeling scheme, displacement ellipsoids drawn at 40% probability level at 170 K for both components disordered around the inversion center. The chemical occupancy of molecules in sites labeled as A and colored orange is 53%, while the occupancy found for site B is 47% and colored green. The crystal packing is shown as viewed along the *a*-axis (*b* and *c* axes are colored green and blue, respectively), and interactions in the crystal packing displaying both disordered components are colored according to donor molecule sites. Molecules in each site are connected through C–H···N interactions. However, molecules found in different sites (A and B) exhibit different bonding patterns with their neighboring molecules. Molecules in site A form antiparallel chains, and molecules in site B form a herringbone pattern. Both sets of molecules form similar C–H···O hydrogen bonds.

To verify that the chemical identity of **1** was preserved, IR spectra of **1a** and **1b** were recorded and directly compared (Figure S10). No significant changes in the spectra were observed except for the signal of the nitrile group (around 2220 cm ^–1^) and a few bands in the fingerprint region (around 880 and 580 cm^–1^) which were observed in **1a** and not in **1b**. It is interesting to note that the signal for the nitrile group is split in **1a**, while it appears as a single peak in **1b**, even though the molecules in **1b** exhibit different packing arrangements. However, the differences in the supramolecular environment around nitrile groups are more pronounced in **1a,** since one set of groups forms supramolecular interactions with neighboring molecules, while another set is oriented toward the channels found in the crystal structure, which are not filled. Nitrile groups in **1b** both form C–H···N bonds, and judging by the relative sharpness of the observed IR band, the interaction energies are comparable and have negligible effects on the carbon–nitrogen bond found in the nitrile group. The position of the N=N stretching of the azoxy bond (around 1460 cm^–1^) coincides in **1a** and **1b**. The chemical identity of **1b** was further confirmed by ^1^H and ^13^C NMR spectroscopy (Figures S1 and S2). Direct comparison of the ^1^H and DEPT-Q ^13^C NMR spectra of **1b** with those of **1a** revealed that the chemical shifts of the aromatic protons and carbons coincide and agree with the previously reported chemical shifts of the azoxy compound 1,2-bis­(4-cyanophenyl)­diazene oxide.^12^ Furthermore, in the ^1^H NMR spectra of **1a** recorded in CDCl_3_ and DMSO-*d*_6_, the signal of residual DCM at 5.32 and 5.76 ppm,^42^ respectively, was detected. Notably, the signal of DCM was not observed in the ^1^H NMR spectra of **1b**. Given that the structure of **1** is preserved during heating, it is still unclear whether **1b** forms directly from **1a** in the solid state by collapsing of the channels or through the gas phase via possible sublimation and desublimation of **1**.

### Synthesis and Characterization of Azoxy-Linked Triazine-Based Polymer (**2**)

Azoxy-linked triazine-based polymer **2** was prepared by the microwave-assisted trifluoromethanesulfonic acid-catalyzed cyclotrimerization of 1,2-bis­(4-cyanophenyl)­diazene oxide (**1**) (Figure 1), adopting the procedure reported in the literature.^25^ The product was collected as a yellowish powder and was insoluble in common organic solvents. In contrast to the FTIR spectrum of the starting compound **1**, the typical C≡N stretching vibration band around 2220 cm^–1^ is absent in the FTIR spectrum of **2** (Figure 4a), indicating the completion of the trimerization reaction. This was further corroborated by the appearance of new bands at 1511 and 1360 cm^–1^, and at 817 cm ^–1^, assigned to the stretching vibrations and the breathing modes of the triazine rings, respectively.^21,25,43^ Furthermore, the FTIR spectrum of **2** showed a band at 1457 cm^–1^ attributed to N=N stretching of the azoxy bond,^44,45^ which coincides with the same vibrational mode in the spectrum of **1**. This confirmed that triazine units are bridged by azoxy bonds in polymer **2**. FTIR data were corroborated by the ^13^C CP/MAS NMR spectrum of **2**, which showed signals at 168.7, 148.4, 145.2, 135.5, 128.8, and 122.5 ppm (Figure 4b). The signal at 168.7 ppm belongs to the carbon atoms inside the triazine ring (labeled as 1 in Figure 4b), signals at 148.4 and 145.2 ppm are attributed to carbon atoms attached to azoxy groups (labeled as 5 and 6, respectively, in Figure 4b), while signals at 135.5, 128.8, and 122.5 ppm are ascribed to aromatic carbons (labeled as 2, 3, and 4, respectively, in Figure 4b). The PXRD pattern of **2**, characterized by a broad peak, revealed its amorphous nature (Figure S11). A similar observation was reported for an analogous azo-linked triazine-based polymer^46,47^ and other covalent triazine frameworks connected by strong covalent bonds, which prevent the formation of ordered structures.^22,25,48,49^

**Figure 4:**
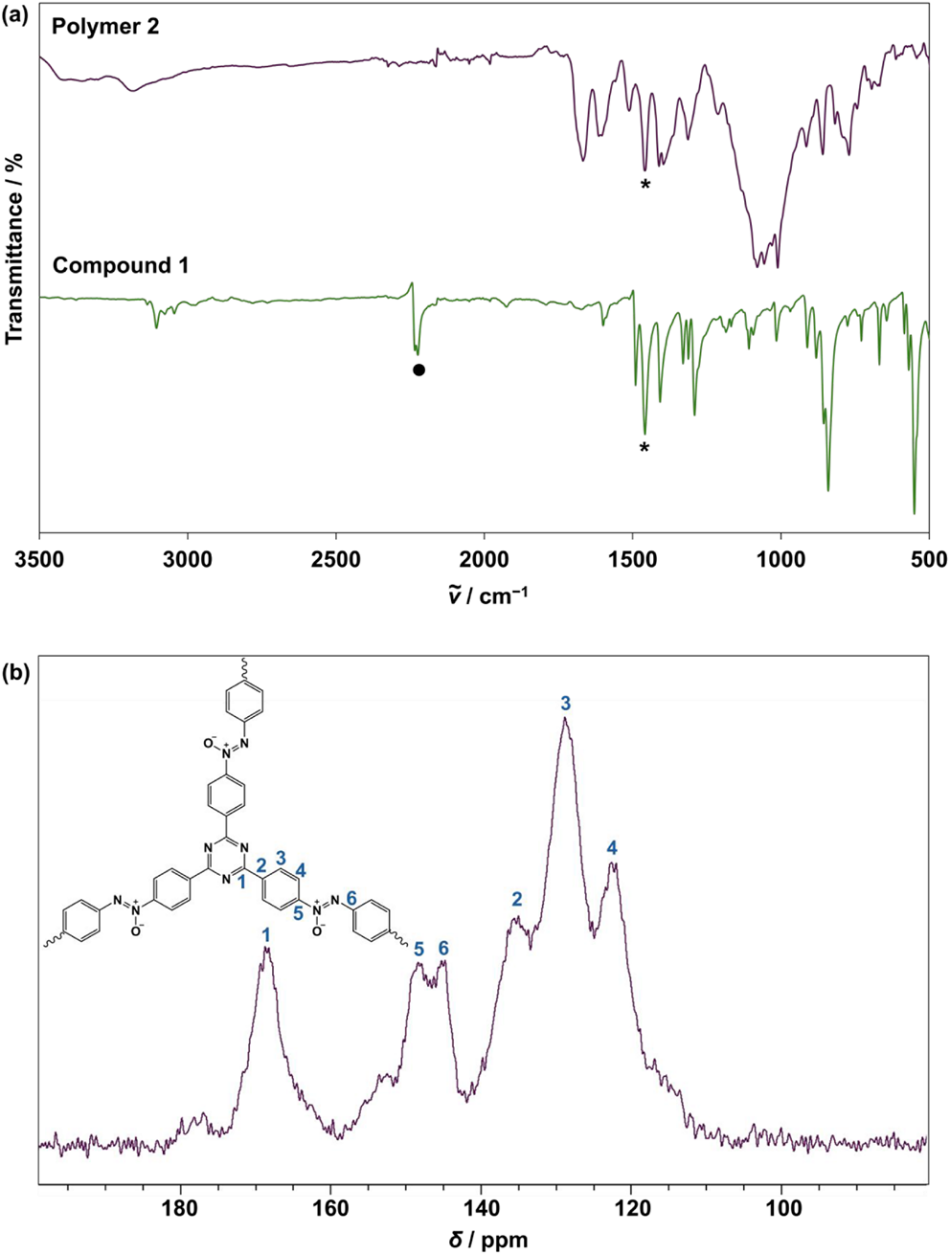
(a) Comparison of FTIR spectra of azoxy-linked triazine-based polymer **2** and 1,2-bis­(4-cyanophenyl)­diazene oxide (**1**). Signals attributed to *N*=N stretching of the azoxy bond are marked with *. Signals attributed to the C≡N stretching vibration band are marked with •. (b) ^13^C CP/MAS NMR spectrum of azoxy-linked triazine-based polymer **2**.

The thermal stability of polymer **2** was determined by TGA. The compound is stable to around 220 °C, after which a slight decrease in weight is observed until around 250 °C, at which point the rate of decomposition slightly increases until 300 °C, when it significantly increases, and a sharp drop in weight is observed until around 370 °C. The sample loses ∼8.0% of its initial weight in the observed thermal event, after which the decomposition rate decreases, and a gradual decrease in weight is observed, with the sample losing an additional ∼2.8% of its initial weight until the end of the isothermal step at 800 °C (Figure S9a). Polymer **2** displays similar thermal stability to its azo-linked counterparts, AZO-T-P2 (synthesized via classical solution synthesis)^46^ and AZO-T-M (synthesized via microwave-assisted synthesis)^47^ which start to decompose at ∼250 °C and ∼190 °C, respectively, and lose less than 10% of their initial weight by 500 °C. However, polymer **2** is much more stable at temperatures higher than 500 °C, since AZO-T-P2 loses around 50% of its initial weight and AZO-T-M loses around 37% of its initial weight by 800 °C. Furthermore, polymer **2** appears to be significantly more thermally stable than its azodioxy-linked counterpart, which also begins to decompose at 220 °C;^26^ however, unlike **2**, it shows a rapid decomposition resulting in a decrease of around 1/3 of its initial weight by 400 °C and more than 50% of its initial weight by 800 °C.

The elemental analysis indicated some differences between the experimentally determined and expected compositions of polymer **2**. Deviation in elemental analysis data from theoretical values is commonly observed in POPs due to incomplete polymerization and adsorption of moisture and gases from air.^20,50–53^

N_2_ adsorption–desorption experiments at 77 K were used to evaluate the porosity of **2**. According to the IUPAC classification,^54^ polymer **2** exhibits a type IV isotherm (Figure S12a), characteristic of mesoporous materials. An H3 hysteresis loop was observed, which is typically associated with materials containing macropores that are not fully filled with the condensed gas. The specific surface area, calculated based on the BET model, is 10.2 m^2^ g^–1^. A low surface area of 4 m^2^ g^–1^ was also observed for the covalent triazine-based framework P1M, synthesized from 1,4-dicyanobenzene by the same synthesis procedure.^25^ When compared with similar polymer networks containing nitrogen–nitrogen linkages, the BET surface area of **2** is lower than that of azo-linked triazine-based polymers AZO-T-P2 (351 m^2^ g^–1^) and AZO-T-P3 (50.8 m^2^ g^–1^), synthesized by conventional heating,^46^ and somewhat higher compared to that of azo-linked triazine polymer AZO-T-M (1.1 m^2^ g^–1^) prepared under microwave conditions,^47^ and triazine-based polymer with azodioxy bridges (4.4 m^2^ g^–1^).^26^ A huge difference between the experimentally determined BET surface area (10.2 m^2^ g^–1^) and the calculated average surface area (1704 m^2^ g^–1^ for the energetically most favorable eclipsed AA configuration)^15^ of **2** was observed. This is somewhat expected since the computational study assumed the formation of a highly ordered AA structure with perfectly aligned neighboring layers, while PXRD analysis revealed the amorphous nature of polymer **2**, resulting in a significantly lower surface area.

The morphology of azoxy-linked triazine-based polymer **2** was examined by SEM. As is evident from the SEM images recorded at different magnifications (Figure 5a–c), polymer **2** features agglomerated particles with sizes in the range of a few tens of μm and a relatively smooth texture. This supports the results of quantitative BET analysis, indicating that **2** is a low-porosity material.

**Figure 5:**
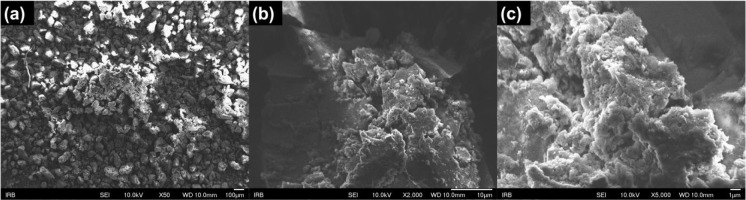
SEM images of azoxy-linked triazine-based polymer **2** at (a) low, (b) medium, and (c) high magnification.

The CO_2_ adsorption performance of **2** was investigated by TGA at approximately 306 K. Surprisingly, the sample did not show an increase in mass when switching to a CO_2_ atmosphere (the observed spikes during cycle switches are experimental artifacts) (Figure S9b). This is contrary to the results of grand canonical Monte Carlo (GCMC) simulations, which predicted a CO_2_ uptake capacity of 20 mg g^–1^ (at 298 K) for a modeled triazine-based framework with azoxy linkages.^15^ Furthermore, the experimentally determined CO_2_ adsorption capacities of the similar azo-linked triazine-based porous polymers synthesized by conventional and microwave heating are 22 and 20 mg g^–1^ (at 306 K), respectively.^47^ An analogous azodioxy-linked polymer also shows some affinity for CO_2_ adsorption of 6 mg g^–1^ (at 306 K),^26^ although its CO_2_ uptake value is significantly lower compared to the triazine polymers bearing azo bridges. The inability of azoxy-bridged triazine polymer **2** to adsorb CO_2_ can primarily be explained by its low surface area. According to previous computational studies, the CO_2_ molecules preferentially bind near the negatively charged oxygens of the azoxy and azodioxy linkages.^15,16^ When compared to the azodioxy-linked system, the number of binding sites near oxygen atoms is halved in the azoxy-linked polymer. This also affects the CO_2_ uptake capacity and could explain the measurable affinity of the azodioxy-linked triazine-based polymer for CO_2_,^26^ even though it possesses a low surface area as its azoxy counterpart **2**.

Besides their application in CO_2_ storage and separation, triazine-based POPs are also explored for their potential as photocatalysts.^22,48,55^ Considering that, we determined the optical band gap (*E*_g_) of **2** by diffuse reflectance spectroscopy. The obtained *E*_g_ value was 2.69 eV (Figure S13), indicating that **2** acts as a wide-bandgap semiconductor. The band gap value of **2** is comparable^25,56–58^ to or somewhat higher^59–61^ than that of similar covalent triazine-based frameworks. Further experimental investigations of the photophysical properties of azoxy-linked triazine polymer **2** are underway.

## Conclusions

In conclusion, we have determined two crystal structures of 1,2-bis­(4-cyanophenyl)­diazene oxide: a dichloromethane solvate (**1a**) with DCM molecules found in channels that run along the *a*-axis and a close-packed form with a highly disordered structure (**1b**) obtained by heating **1a** to at least 190 °C in a N_2_ atmosphere. It was shown by SCXRD measurements that **1a** retains its crystal structure up to at least 127 °C, and TGA measurements reveal it is stable up to ∼190 °C. At this point, DCM molecules leave the structure, and a close-packed form **1b** forms. The mechanism of transformation from **1a** to **1b** at ∼190 °C is still unclear; however, IR and NMR measurements confirmed that the chemical structure of **1** is preserved. The molecular structure of **1** was accurately determined since the molecule does not display disorder in **1a**. Form **1b** shows heavy disorder, and it was not possible to model molecules of **1** without restraints. This trend is consistent throughout the whole series of aromatic azoxy structures, as CSD searches revealed that only 43% of the obtained hits correspond to crystal structures that are not disordered. Furthermore, it shows the importance of good-quality crystal structures and data for the accurate determination of molecular structures.

The nitrile-substituted azoxybenzene derivative **1** was further used for the synthesis of azoxy-linked triazine polymer **2**, which was suggested by a previous computational chemistry study as a potential CO_2_ adsorbent. Microwave-assisted trifluoromethanesulfonic acid-catalyzed cyclotrimerization of **1** produced an amorphous solid with good thermal stability that was insoluble in common organic solvents. Polymer **2** exhibits a low BET surface area of 10.2 m^2^ g^–1^, which is consistent with the morphology observed via SEM analysis. On the contrary to previous GCMC predictions, thermogravimetric gas sorption analysis at 306 K indicated that azoxy-linked triazine polymer **2** does not adsorb CO_2_, which can be primarily attributed to its low porosity. Notably, preliminary measurements showed that polymer **2** exhibits an optical band gap of 2.69 eV, revealing its potential as a wide-bandgap semiconductor material. Further studies to explore the photophysical properties of **2** are currently underway.

## Supplementary Material







## Notes

Synthesis procedures and characterization data for compounds **1** and **2**; NMR spectra of compounds **1a** and **1b**; single-crystal X-ray crystallography data of compounds **1a** and **1b**; powder X-ray crystallography data of compounds **1a** and **1b**; thermogravimetric analysis of compounds **1a** and polymer **2**; IR spectra of compounds **1a** and **1b**; powder X-ray crystallography data of polymer **2**; N_2_ adsorption–desorption analysis of polymer **2**; diffuse reflectance spectroscopy data of polymer **2** (PDF)
Crystallographic data for molecule **1** (CIF)
Crystallographic data for molecule **2** (CIF)
