# Supplementary material for: Structural Characterization of 1,2-Bis(4-cyanophenyl)diazene Oxide and Its Cyclotrimerization to a Triazine-Linked Polymer
Source: ACS Omega. 2026 Jul 29;11(31):45708–16. doi: 10.1021/acsomega.5c11736 (PMC13470967; doi:10.1021/acsomega.5c11736)
Supplement: Supplementary file 1 [file ao5c11736_si_001.pdf]

Supplementary Materials

Structural characterization of 1,2-bis(4-cyanophenyl)diazene oxide and its cyclotrimerization to a triazine-linked polymer

Mladen Borovina, Vesna Petrović Peroković, Barbara Panić, Ivana Biljan*

Department of Chemistry, University of Zagreb Faculty of Science, Horvatovac 102a, HR-10000 Zagreb, Croatia

1. Synthesis and characterization of compounds **1** and **2**

Synthesis of 1,2-bis(4-cyanophenyl)diazene oxide (1)

During the previously described synthesis of 4-nitrosobenzonitrile,[i] which was prepared by oxidation of 4-aminobenzonitrile (1 g, 8.5 mmol) with Oxone at room temperature in a DCM-water mixture, compound **1** was obtained as a by-product and isolated by column chromatography using DCM as the solvent as a yellow solid (120 mg, 11%). R_f = 0.54 (DCM). ^{1}H NMR ($CDCl_3$, 400 MHz) δ/ppm: 8.48 (d, J = 8.8 Hz, 2H), 8.25 (d, J = 8.7 Hz, 2H), 7.89 (d, J = 8.8 Hz, 2H), 7.82 (d, J = 8.7 Hz, 2H). ^{1}H NMR (DMSO-d_6, 400 MHz) δ/ppm: 8.45 (d, J = 8.5 Hz, 2H), 8.18−8.14 (m, 4H), 8.10 (d, J = 8.4 Hz, 2H). ^{13}C NMR ($CDCl_3$, 100 MHz) δ/ppm: 150.3 (C—N(O)=N), 146.3 (C—N=N(O)), 133.1 (CH × 2), 132.9 (CH × 2), 126.1 (CH × 2), 123.4 (CH × 2), 118.3 (C≡N), 117.4 (C≡N), 116.2 (C-C≡N), 113.1 (C-C≡N). IR (ATR) $\tilde{\nu}$ / cm^{-1}: 3105, 2223, 1599, 1489, 1458, 1407, 1291, 840. Elemental analysis: 66.74 %C (calc. 67.74 **1**; 62.11 **1a**); 3.79 %H (calc. 3.25 **1**; 3.16 **1a**); 21.82 %N (calc. 22.57 **1**; 20.20 **1a**).

a)

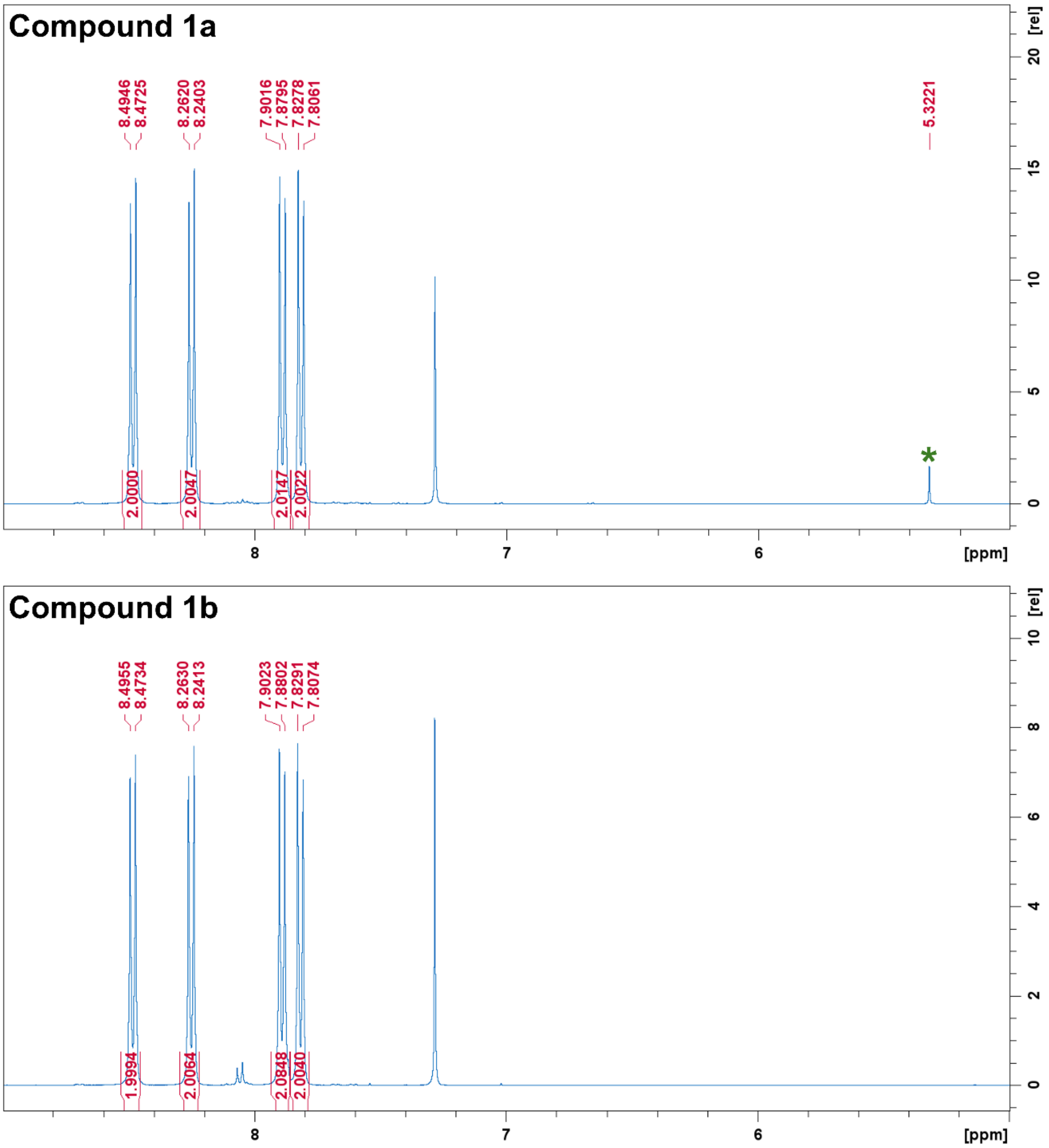

b)

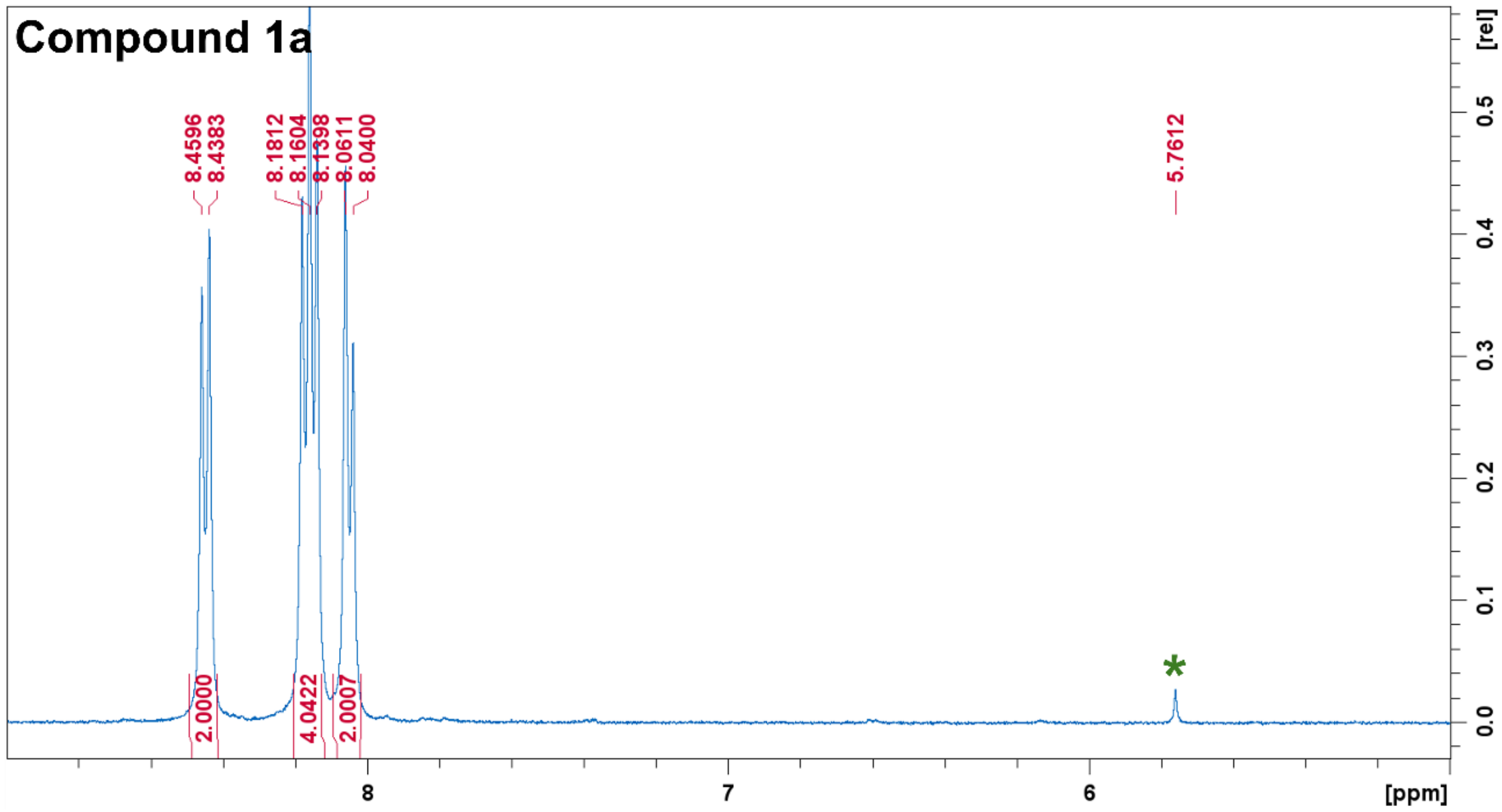

Compound 1a
8.4596
8.4383
8.1812
8.1604
8.1398
8.0611
8.0400
5.7612
*
2.0000
4.0422
2.0007
[rel]
0.5
0.4
0.3
0.2
0.1
0.0
8
7
6
[ppm]

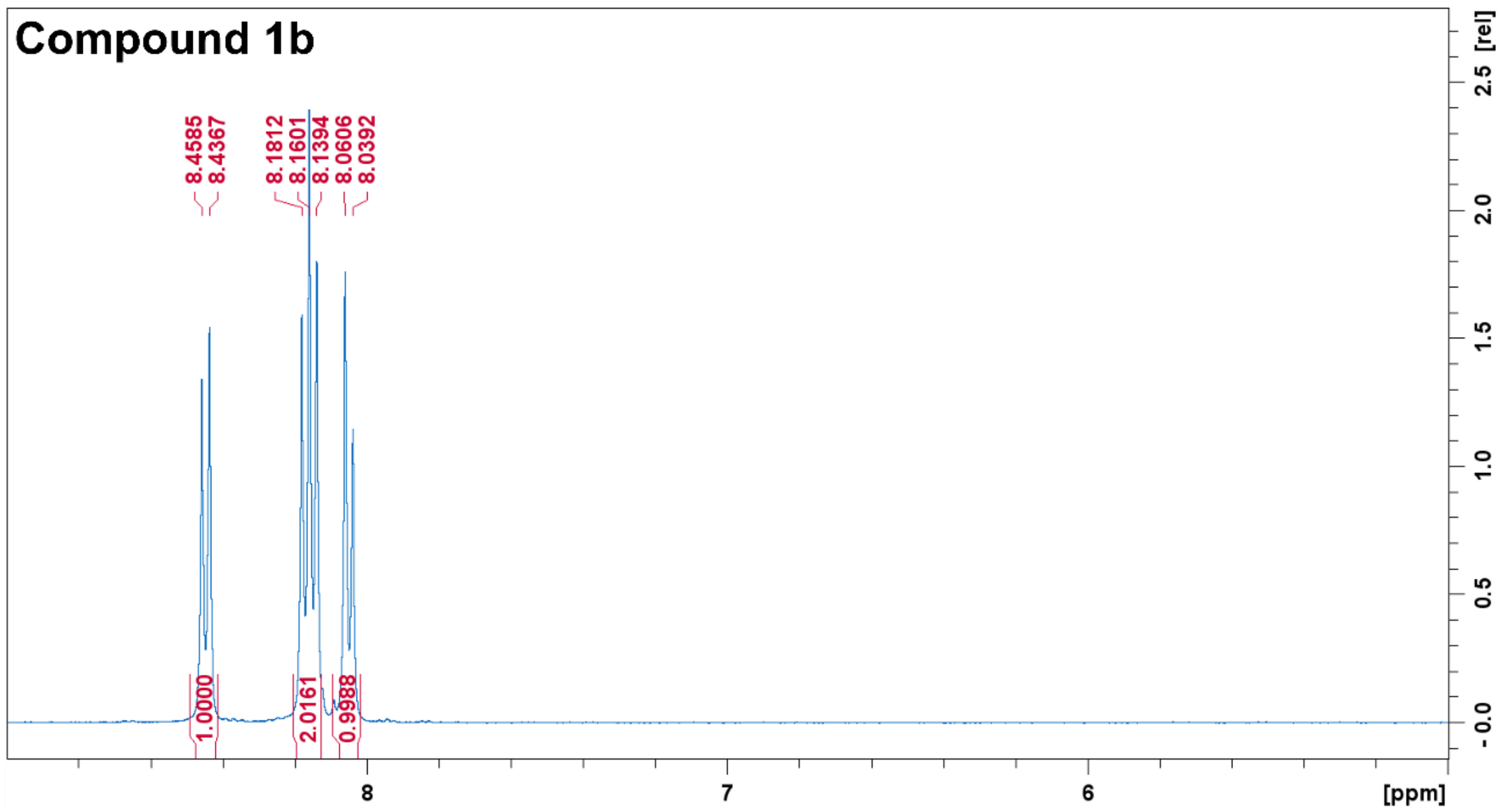

Compound 1b
8.4585
8.4367
8.1812
8.1601
8.1394
8.0606
8.0392
1.0000
2.0161
0.9988
[rel]
2.5
2.0
1.5
1.0
0.5
-0.0
8
7
6
[ppm]

c)

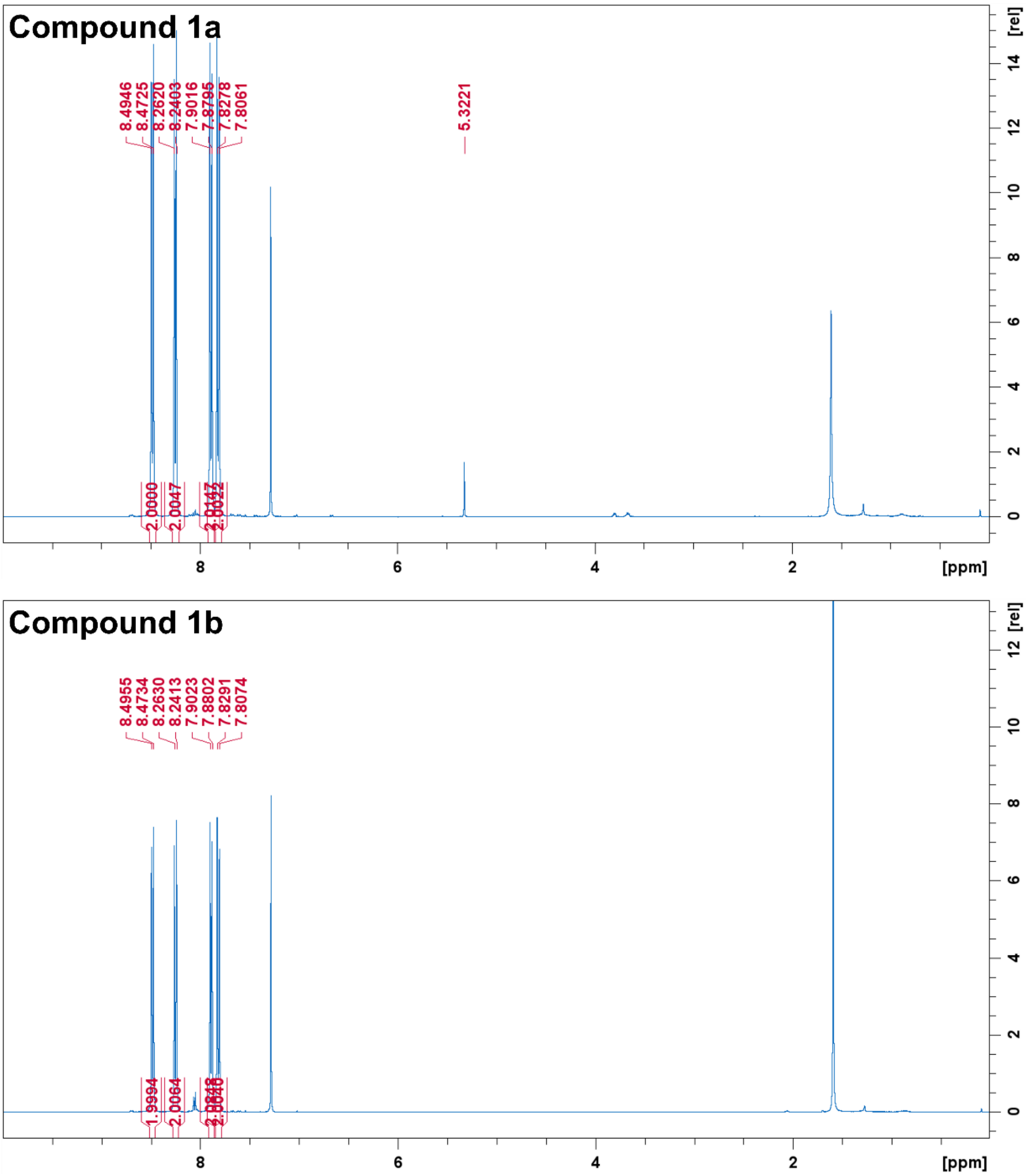

Compound 1a
8.4946
8.4725
8.2620
8.2403
7.9016
7.8795
7.8278
7.8061
5.3221
2.0000
2.0047
[rel]
[ppm]
Compound 1b
8.4955
8.4734
8.2630
8.2413
7.9023
7.8802
7.8291
7.8074
1.9994
2.0064
[rel]
[ppm]

d)

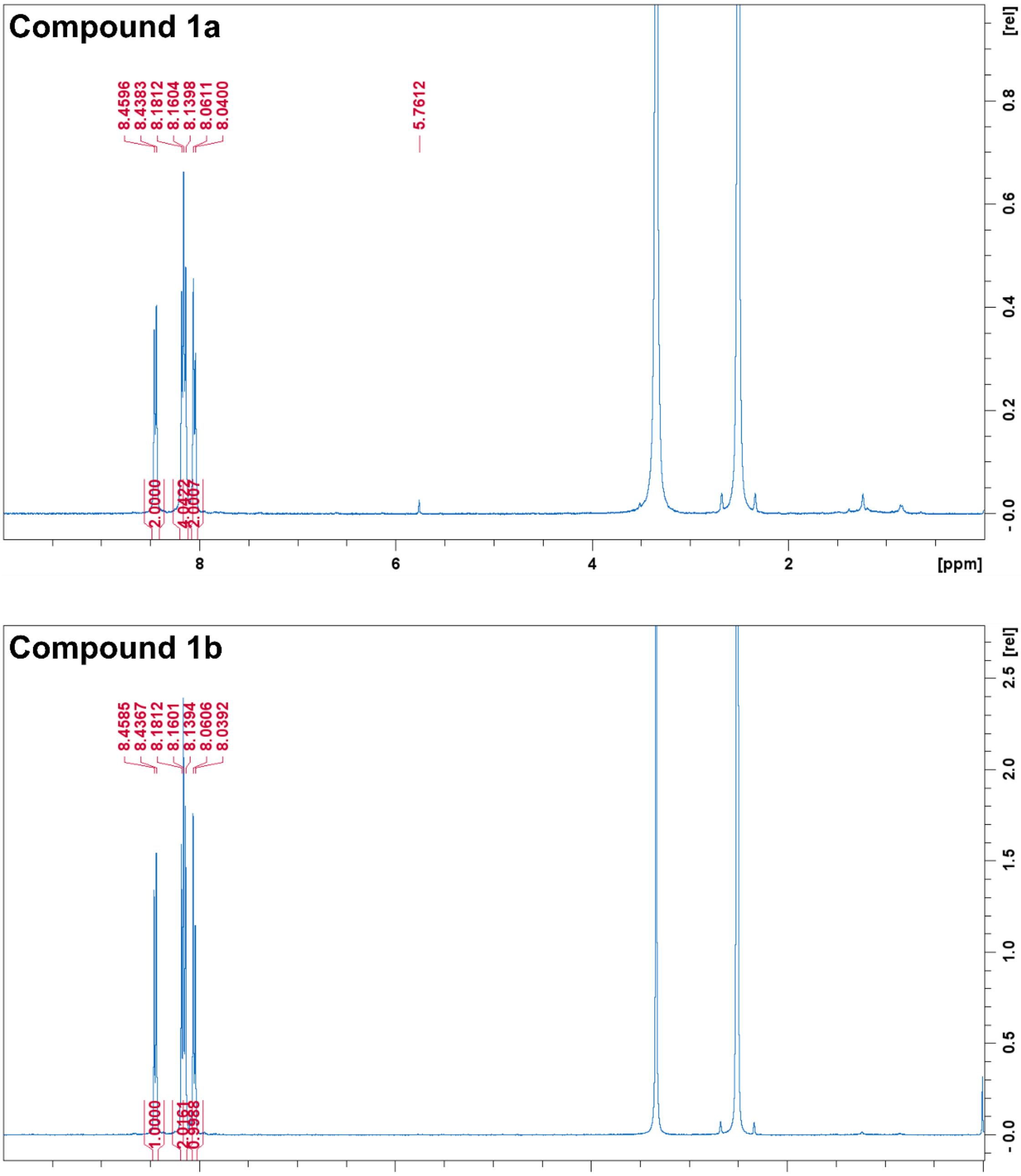

Figure S1. Comparison of ^{1}H NMR spectra of compounds **1a** and **1b** in (a) $CDCl_3$ and (b) DMSO-d_6. The signal marked with * corresponds to dichloromethane (DCM). Comparison of full-range ^{1}H NMR spectra of compounds **1a** and **1b** in (c) $CDCl_3$ and (d) DMSO-d_6.

a)

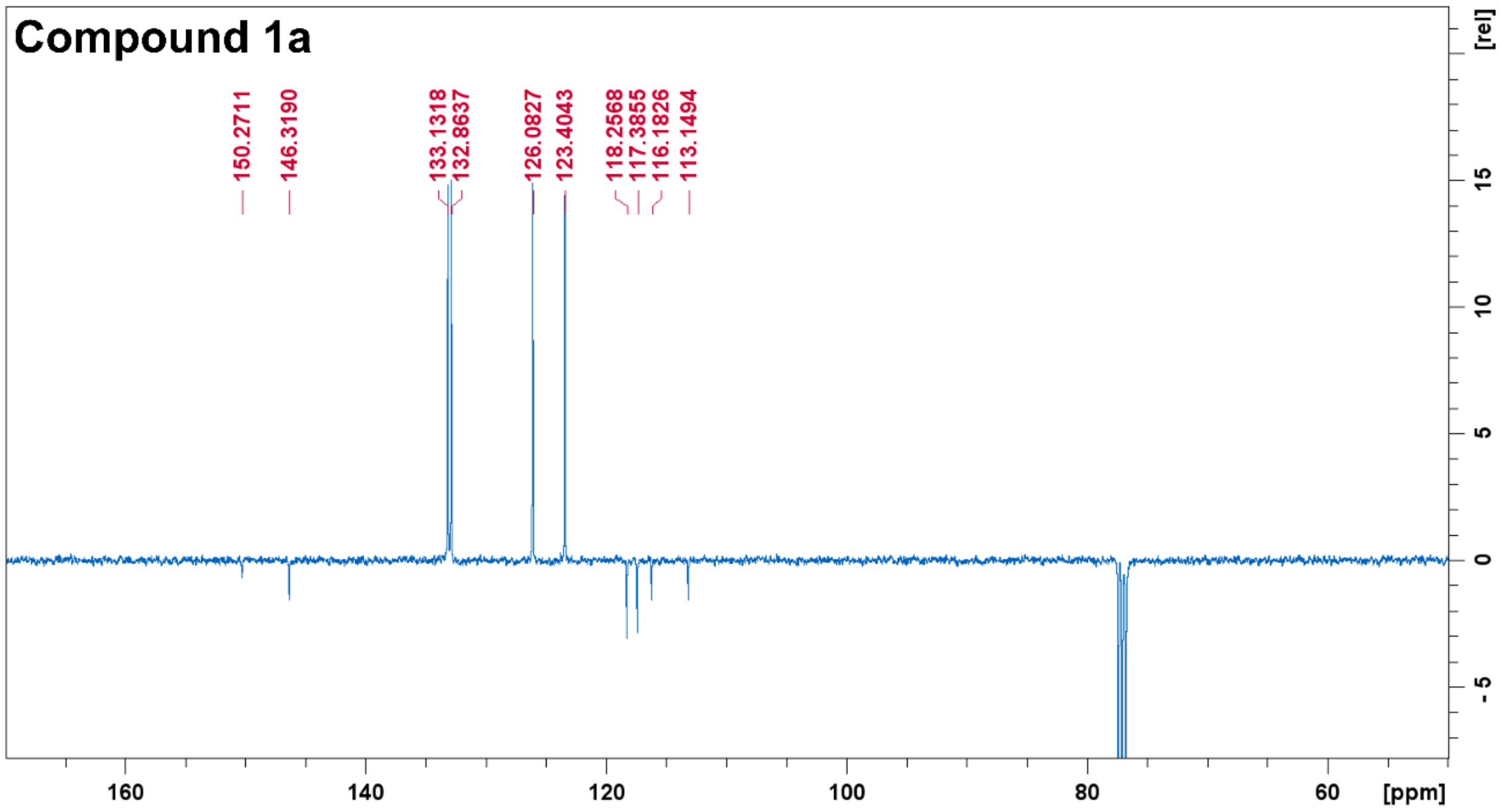

Compound 1a
150.2711
146.3190
133.1318
132.8637
126.0827
123.4043
118.2568
117.3855
116.1826
113.1494
[rel]
160
140
120
100
80
60
[ppm]

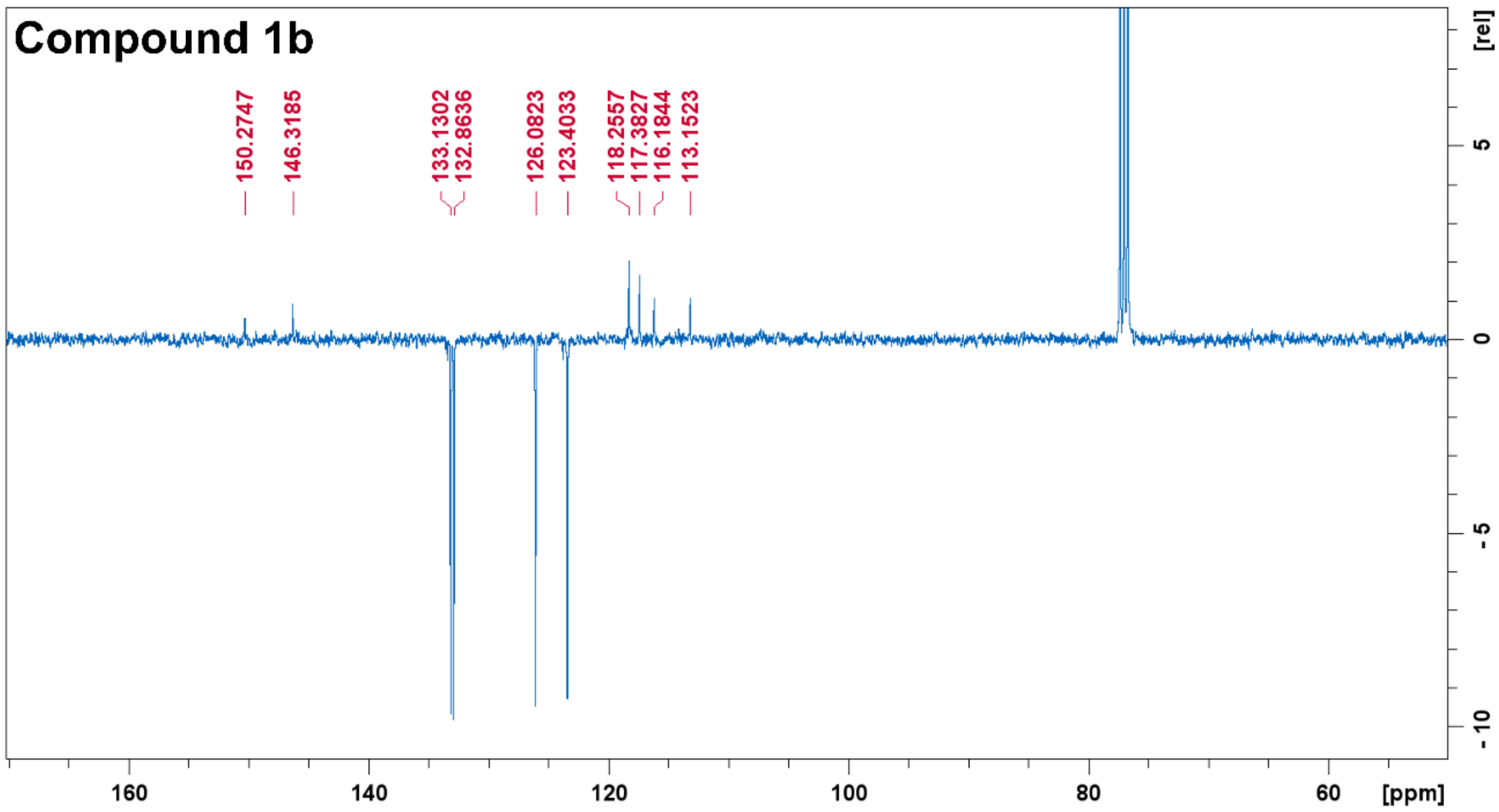

Compound 1b
150.2747
146.3185
133.1302
132.8636
126.0823
123.4033
118.2557
117.3827
116.1844
113.1523
[rel]
160
140
120
100
80
60
[ppm]

b)

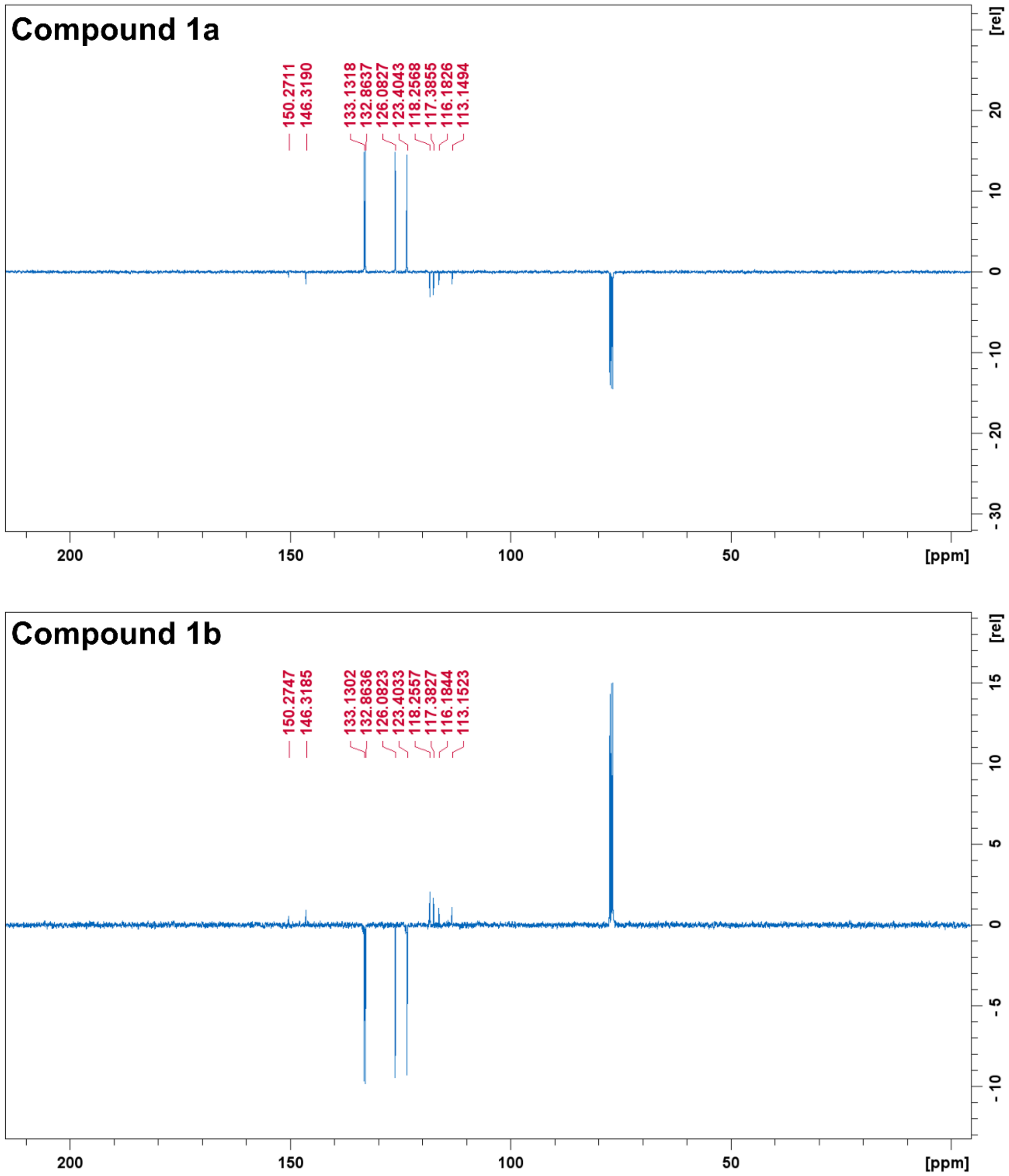

Figure S2. Comparison of (a) DEPT-Q ^{13}C NMR spectra of compounds **1a** and **1b** in $CDCl_3$ and (b) full-range DEPT-Q ^{13}C NMR spectra of compounds **1a** and **1b** in $CDCl_3$.

Synthesis of azoxy-linked triazine-based polymer (2)

Polymer **2** was prepared under microwave synthesis conditions according to the previously described procedure.[ii] Compound **1** (100 mg, 0.403 mmol) and trifluoromethanesulfonic acid (1 mL) were added to a 10 mL Pyrex pressure vessel. The reaction mixture was stirred and heated at 300 W to 110 °C for 30 min. To the resulting red-brown solution, 0.1 M ammonia solution was added with cooling. The obtained precipitate was filtered and processed according to the described literature procedure.[ii] An amorphous yellowish product **2** (54 mg, 60%) was obtained and characterized by FTIR and solid-state

^{13}C CP/MAS NMR spectroscopy. IR (ATR) $\tilde{\nu}$ / cm^{-1}: 3182, 1666, 1603, 1511, 1457, 1410, 1395, 1360, 1312, 1056, 858, 817, 770. ^{13}C CP/MAS NMR (400 MHz) δ / ppm: 168.7, 148.4, 145.2, 135.5, 128.8, 122.5. Elemental analysis: 55.49 %C (calc. 65.62); 3.83 %H (calc. 4.20); 17.35 %N (calc. 21.86).

2. Single crystal X-ray crystallography

CCDC Nos 2475649 and 2475650 contain supplementary crystallographic data. Data can be obtained free of charge from the Cambridge Crystallographic Data Centre via https://www.ccdc.cam.ac.uk/structures/ .

Structure refinement of 1a.

Disordered dichloromethane molecules were treated using idealized fragments from I. Guzei's fragment library,[iii] which were fitted in Olex-2. Carbon chlorine distances were restrained to 1.771 Å using *DFIX* and chlorine chlorine distances to 2.916 Å using *DANG* restraints. The occupancy of non-hydrogen atoms in dichloromethane molecules was linked to free variables and refined. Non-hydrogen atoms were refined anisotropically except for disordered solvent molecules, which were refined isotropically. All hydrogen atoms were placed in calculated positions based on geometry.

Structure refinement 1b.

Disorder was treated using *SAME* restraints for all atoms and *FLAT* restraints for the aromatic ring and nitrile group. Oxygen nitrogen distances were treated with *DFIX* restraints, and distances between symmetry equivalent nitrogen atoms with *SADI* restraints. The occupancy of non-hydrogen atoms was linked to a free variable and refined. All non-hydrogen atoms were refined anisotropically, and all hydrogen atoms were placed in calculated positions based on geometry.

Table S1. Crystallographic data for compounds **1a** and **1b**.

**Compound**	**1a**	**1b**
Formula	$C_{14}H_8N_4O$, 0.341(CH_2Cl_2)	$C_{14}H_8N_4O$
M_r	277.30	248.24
Colour and habit	Orange rod	Yellow plate
Crystal dimensions (mm^3)	0.045 x 0.068 x 0.614	0.05 x 0.17 x 0.21
Crystal system	Triclinic	Monoclinic
Space group	$P\bar{1}$	$P 2_1/c$
a (Å)	3.86680(10)	4.67360(10)
b (Å)	12.4582(3)	10.2060(2)
c (Å)	14.7933(3)	12.6689(4)
α (°)	107.048(2)	90
β (°)	94.044(2)	98.156(3)
γ (°)	97.226(2)	90
V (Å^3)	671.57(3)	598.18(3)
Z	2	2
Temperature (K)	296	170
D_{calc} (g cm^{-3})	1.371	1.378
Radiation wavelength *λ* (Å), *μ* (mm^{-1})	Cu *Kα* 1.54184, 1.955	Cu *Kα* 1.54184, 0.756
F (000)	285	256
Scan type	Ω	Ω
h, *k*, *l* range	–3:4, –15:15, –18:18	–5:5, –12:12, –15:13
θ range/°	3.1 - 77.0	5.59 – 76.56
No. measured reflections	12727	5946
No. Independent reflections	2727	1225
No. Observed reflections, $I \geq 2\sigma$	2261	1023
Completeness	0.964	0.977
No. refined parameters	198	186
R_{int}	0.034	0.029
R, *wR* [$I \geq 2\sigma$]	0.0624, 0.1995	0.0367, 0.0963
R, *wR* [all data]	0.0690, 0.2071	0.0437, 0.1017
Goodness of fit on F^2, *S*	1.086	1.043
Max, min. El. density (e Å^{-3})	–0.308, 0.367	–0.204, 0.107
CCDC number	2475649	2475650

Table S2. Selected bond lengths and angles for compounds **1a** and **1b**.

Compound							
**1a**				**1b**			
Bond	*d* / Å	Bond	*d* / Å	Bond	*d* / Å	Bond	*d* / Å
O1 – N1	1.255(2)			O1A–N1A	1.472(5)	O1B–N1B	1.471(6)
N1 – N2	1.265(2)			N1A–N1A^{i}	1.305(8)	N1B–N1B^{i}	1.286(9)
C1 – N1	1.473(3)	C8 – N2	1.413(3)	C1A–N1A	1.453(7)	C1B–N1B	1.442(8)
C1 – C2	1.379(3)	C8 – C9	1.398(3)	C1A–C2A	1.356(6)	C1B–C2B	1.360(6)
C2 – C3	1.377(3)	C9 – C10	1.375(3)	C2A–C3A	1.398(8)	C2B–C3B	1.400(9)
C3 – C4	1.393(3)	C10 – C11	1.375(3)	C3A–C4A	1.372(9)	C3B–C4B	1.380(10)
C4 – C5	1.385(3)	C11 – C12	1.388(3)	C4A–C5A	1.365(9)	C4B–C5B	1.377(10)
C5 – C6	1.376(3)	C12 – C13	1.366(3)	C5A–C6A	1.369(7)	C5B–C6B	1.368(8)
C6 – C1	1.378(3)	C13 – C8	1.388(3)	C6A–C1A	1.374(5)	C6B–C1B	1.380(6)
C4 – C7	1.437(3)	C11 – C14	1.436(3)	C4A–C7A	1.442(8)	C4B–C7B	1.443(9)
C7 – N3	1.144(3)	C14 – N4	1.140(3)	C7A–N2A	1.137(8)	C7B–N2B	1.133(9)

1a		**1b**			
Angle	∠ / °	Angle	∠ / °	Angle	∠ / °
O1 – N1– N2	128.5(2)	O1A – N1A– N1A^{i}	131.7(8)	O1B – N1B– N1B^{i}	131.0(9)
O1 – N1– C1	117.1(2)	O1A – N1A – C1A	112.2(4)	O1B – N1B – C1B	113.2(5)
N2 – N1– C1	114.4(2)	N1A^{i} – N1A – C1A	115.7(8)	N1B^{i} – N1B – C1B	115.7(9)
N1 – N2– C8	120.0(2)				
C4 – C7 –N3	179.1(2)	C4A – C7A – N2A	178.5(10)	C4B – C7B – N2B	178.7(10)
C11 – C14 – N4	179.0(3)				

Symmetry operators: 1 – *x*, 1 – *y*, 1 – *z* (i)

Table S3. Unit cell parameters of **1a** determined at different temperatures.

T / K	*a* / Å	*b* / Å	*c* / Å	*α* / °	*β* / °	*γ* / °	*V* / Å^3
**274**	3.8545(17)	12.477(5)	14.754(2)	107.13(2)	94.31(2)	97.04(4)	668.3(4)
**300**	3.870(2)	12.460(7)	14.807(8)	107.08(5)	94.03(5)	97.11(5)	672.9(6)
**330**	3.901(3)	12.390(12)	14.875(12)	106.87(8)	93.53(7)	97.35(8)	679(1)
**360**	3.913(2)	12.392(6)	14.923(9)	106.63(5)	93.11(5)	98.01(5)	683.3(7)
**380**	3.927(2)	12.362(7)	14.971(8)	106.53(5)	92.67(5)	98.34(5)	686.4(7)
**390**	3.931(4)	12.40(4)	14.982(15)	106.31(17)	92.40(9)	98.86(16)	690(3)
**400**	3.952(2)	12.296(7)	15.010(9)	106.27(5)	92.16(5)	98.90(5)	689.2(7)

Table S4. Geometric parameters of hydrogen and halogen bonds in **1a** and **1b**.

**1a**				
Interaction	*d*(D···A) / Å	*d*(H···A) / Å	∠(D–H···A) / °	Sym. operator on A
C6 – H6 ··· O1	3.445(3)	2.64	145	–*x*, 1−*y*, 1−*z*
C5 – H5 ··· N3	3.525(3)	2.71	147	–*x*, 2−*y*, 1−*z*
C10 – H10 ··· N3	3.463(3)	2.77	132	1+*x*, *y*−1, *z*

**1b**				
Interaction	*d*(D···A) / Å	*d*(H···A) / Å	∠(D–H···A) / °	Sym. operator on A
C3A – H3A ··· O1A	3.366(9)	2.67	131	*x*−1, 0.5−*y*, *z*−0.5
C5A – H5A ··· N2A	3.224(10)	2.35	153	–*x*−1, –*y*, 1−*z*
C5B – H5B ··· O1B	3.167(10)	2.49	129	–*x*, *y*−0.5, 0.5−*z*
C5B – H5B ··· N2B	3.190(12)	2.72	111	–*x*−1, *y*+0.5, 0.5−*z*
C6B – H6B ··· N2B	3.179(12)	2.67	113	–*x*−1, *y*+0.5, 0.5−*z*

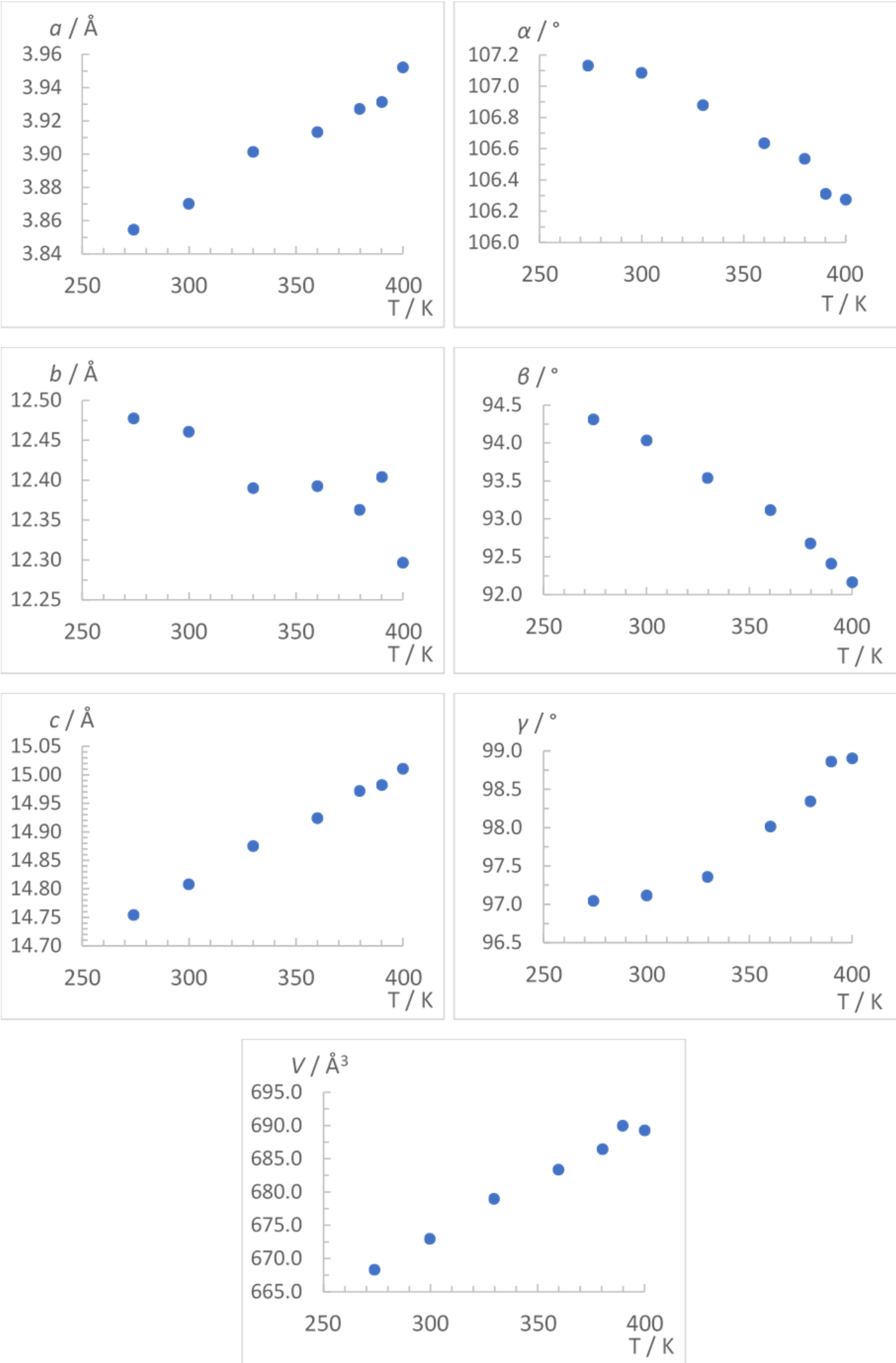

Figure S3. Unit cell parameters of **1a** determined at different temperatures from 273 K to 400 K.

3. Powder X-ray diffraction (PXRD) of 1,2-bis(4-cyanophenyl)diazene oxide

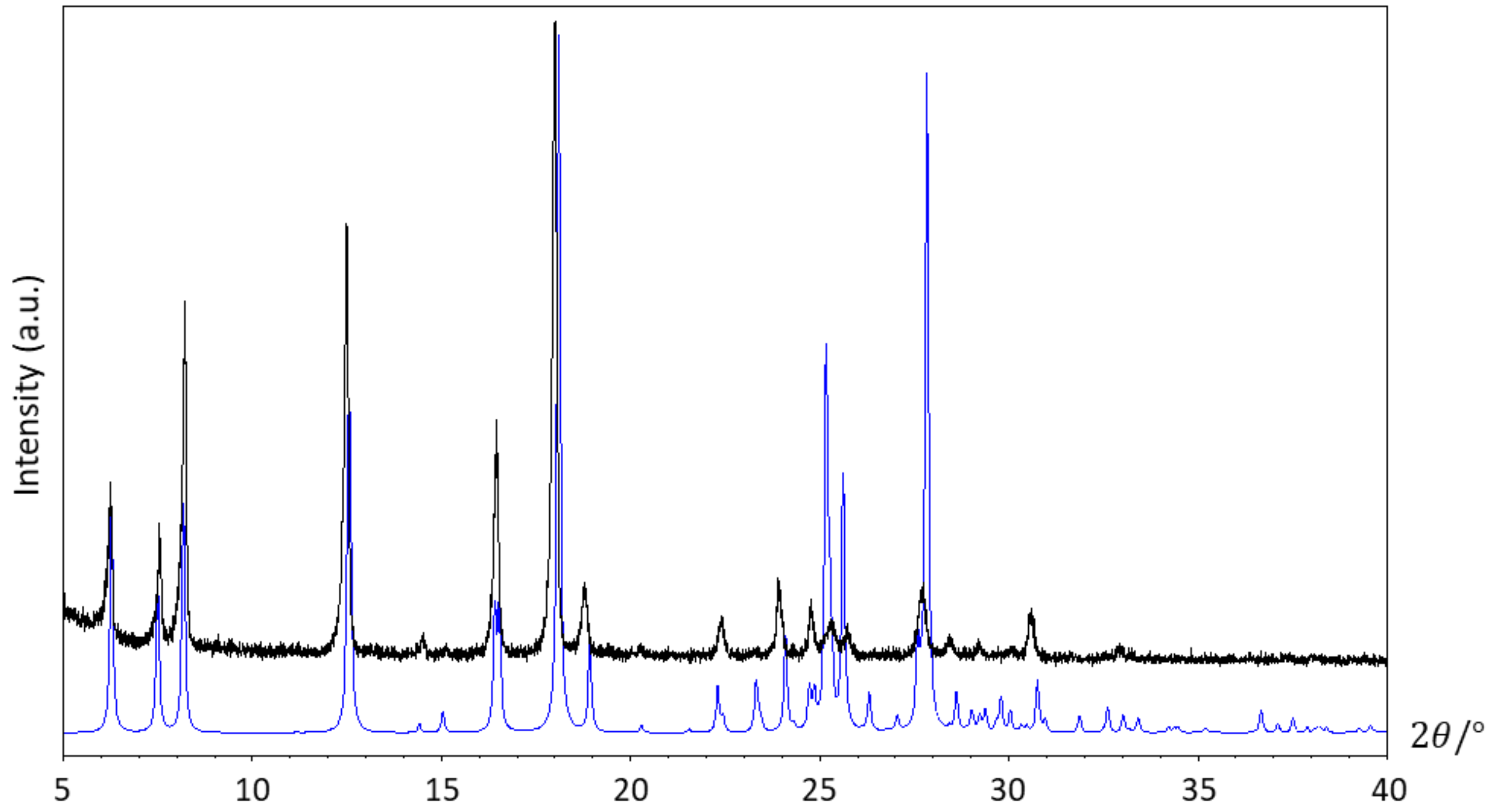

Figure S4. Experimental (black) and calculated (blue) PXRD pattern of **1a**.

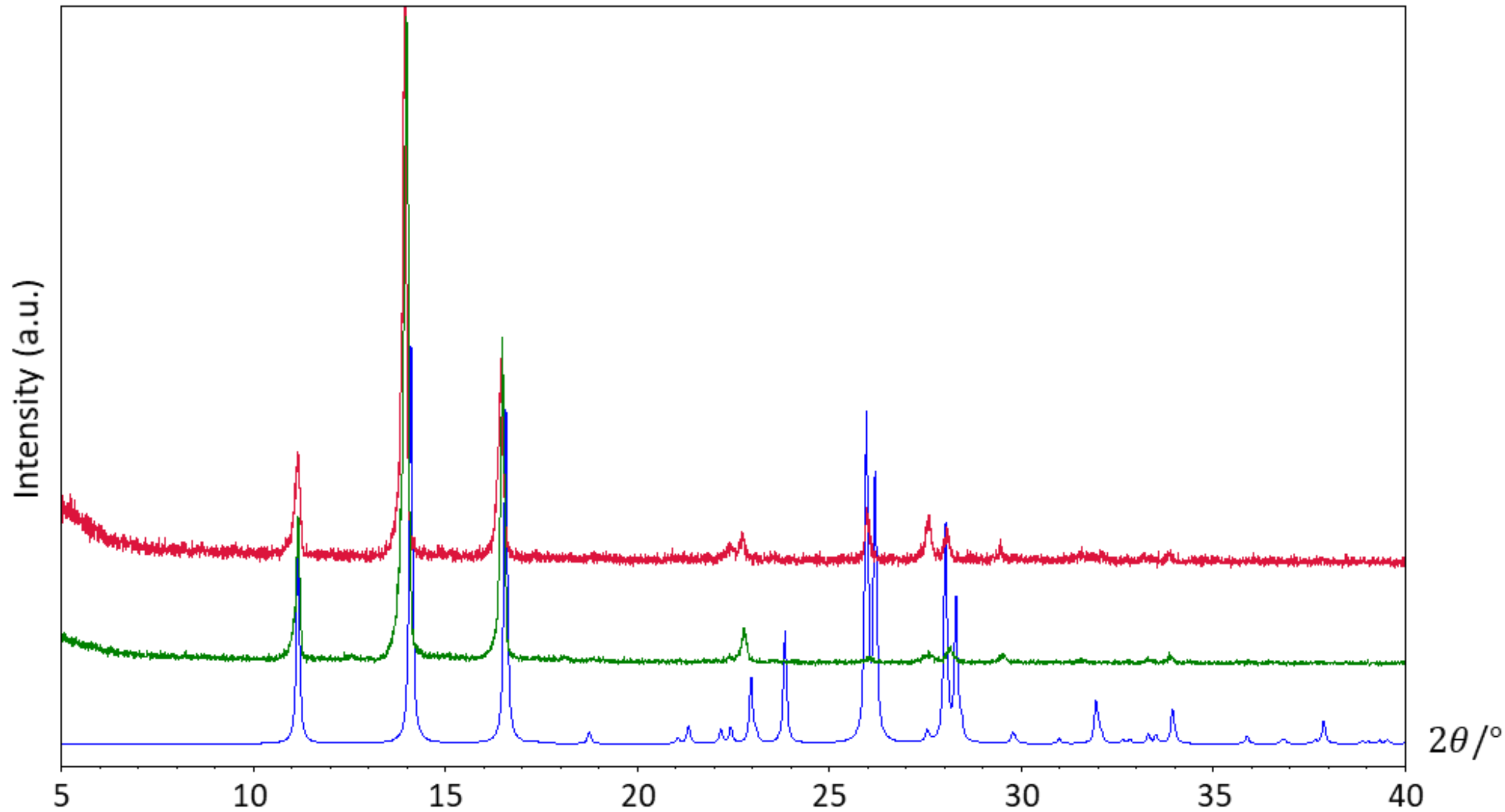

Figure S5. Experimental PXRD traces of **1b** obtained by heating **1a** to 190 °C (green) and 210 °C (red), calculated (blue) PXRD pattern of **1b** (data collected at –103 °C).

4. Thermogravimetric analysis

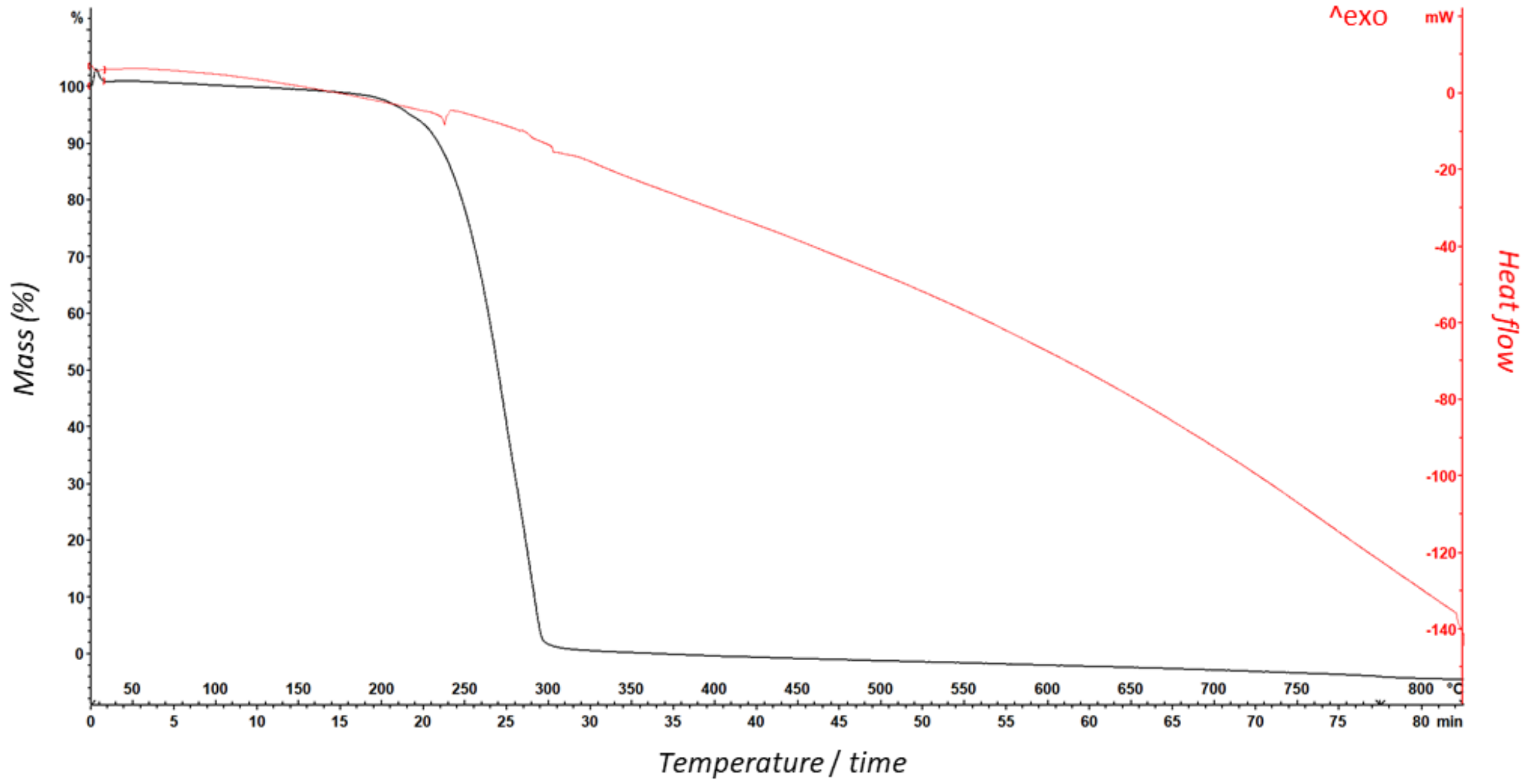

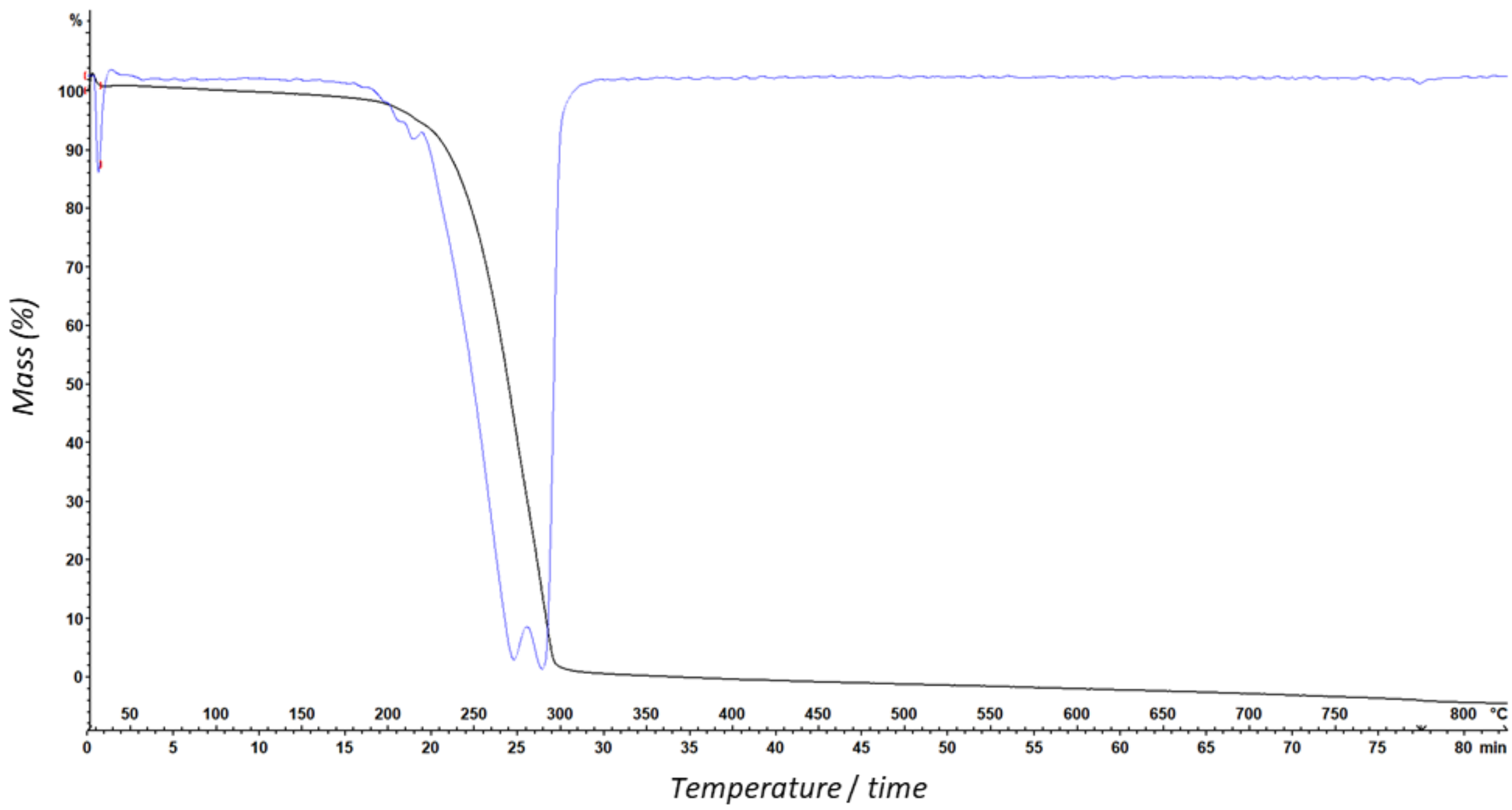

Figure S6. TGA (black), DSC (red), and 1st derivative of weight (blue) curves of **1a** heated from 25 to 800 °C at a heating rate of 10 °C min^{-1} under a nitrogen atmosphere.

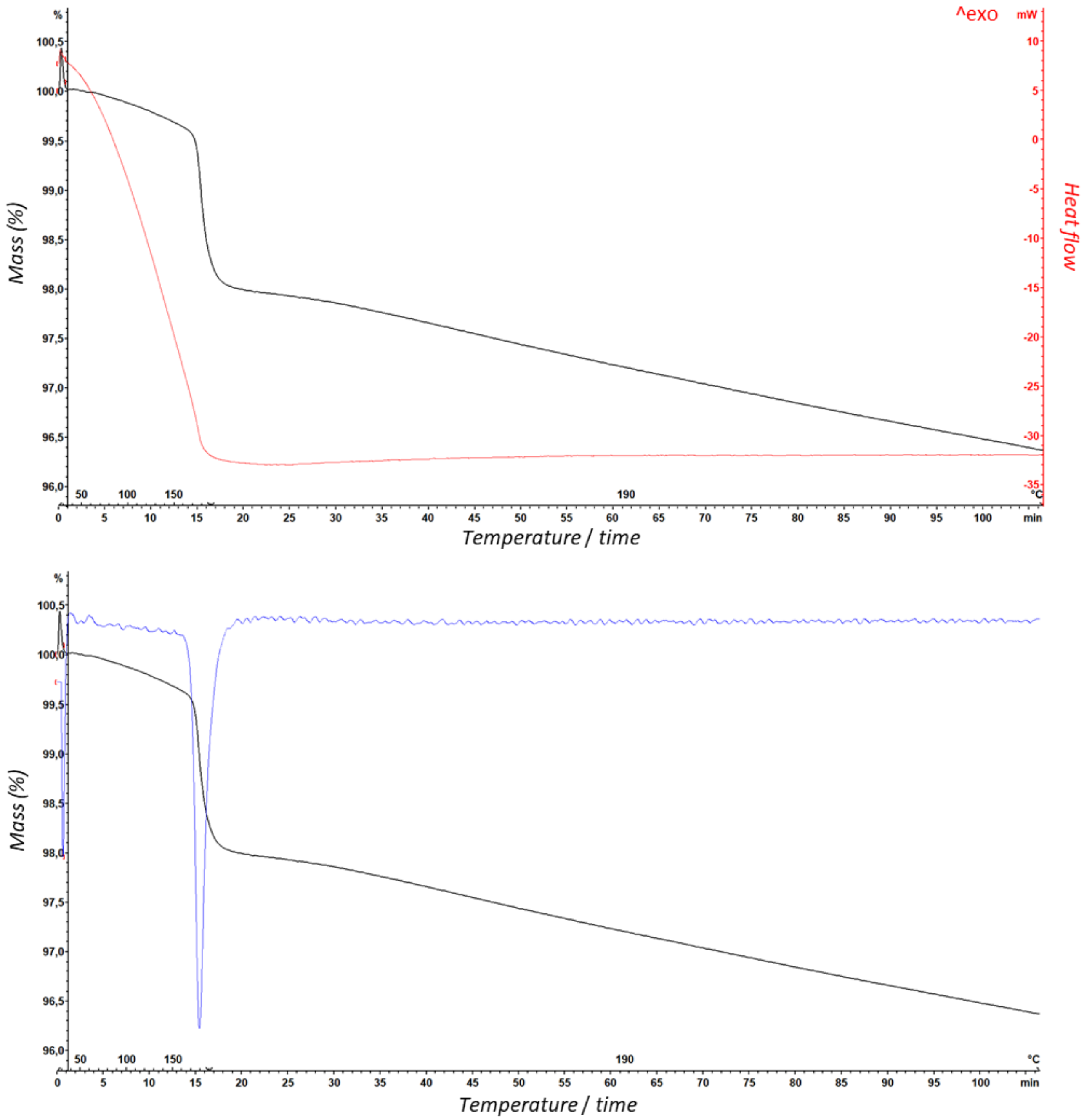

Figure S7**.** TGA (black), DSC (red), and 1st derivative of weight (blue) curves of **1a** heated from 25 to 190 °C at a heating rate of 10 °C min^{-1} under a nitrogen atmosphere.

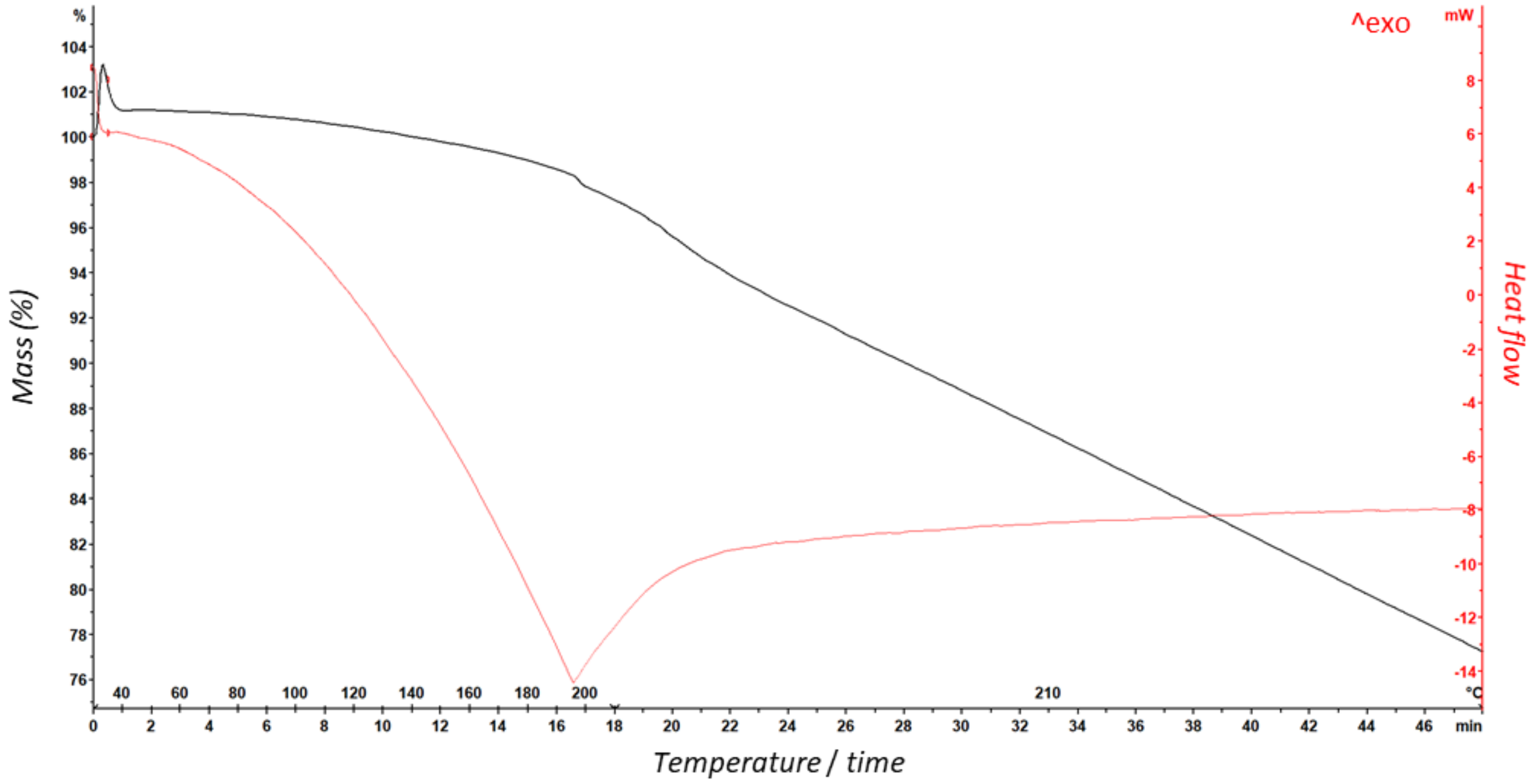

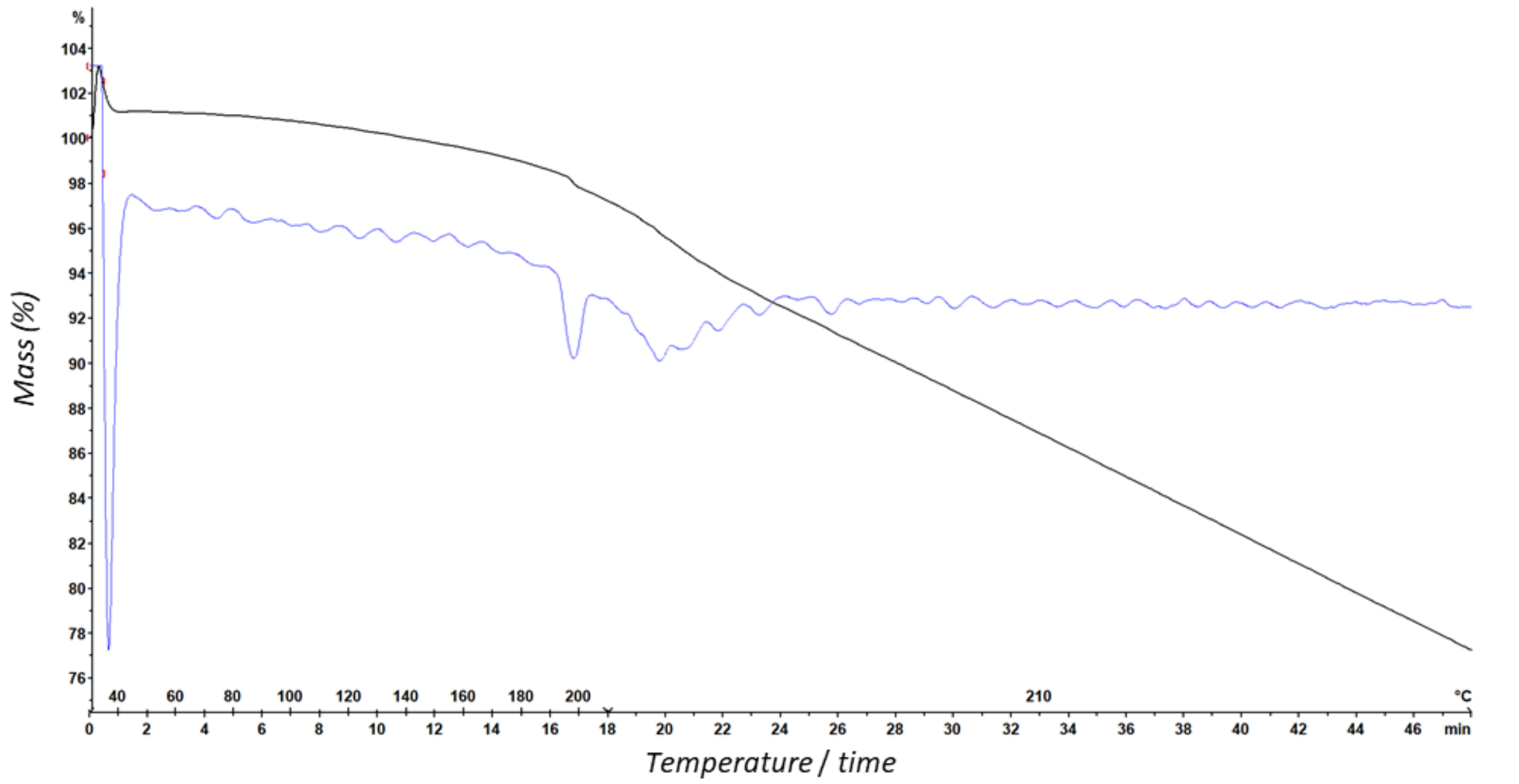

Figure S8. TGA (black), DSC (red), and 1st derivative of weight (blue) curves of **1a** heated from 30 to 210 °C at a heating rate of 10 °C min^{-1} under a nitrogen atmosphere.

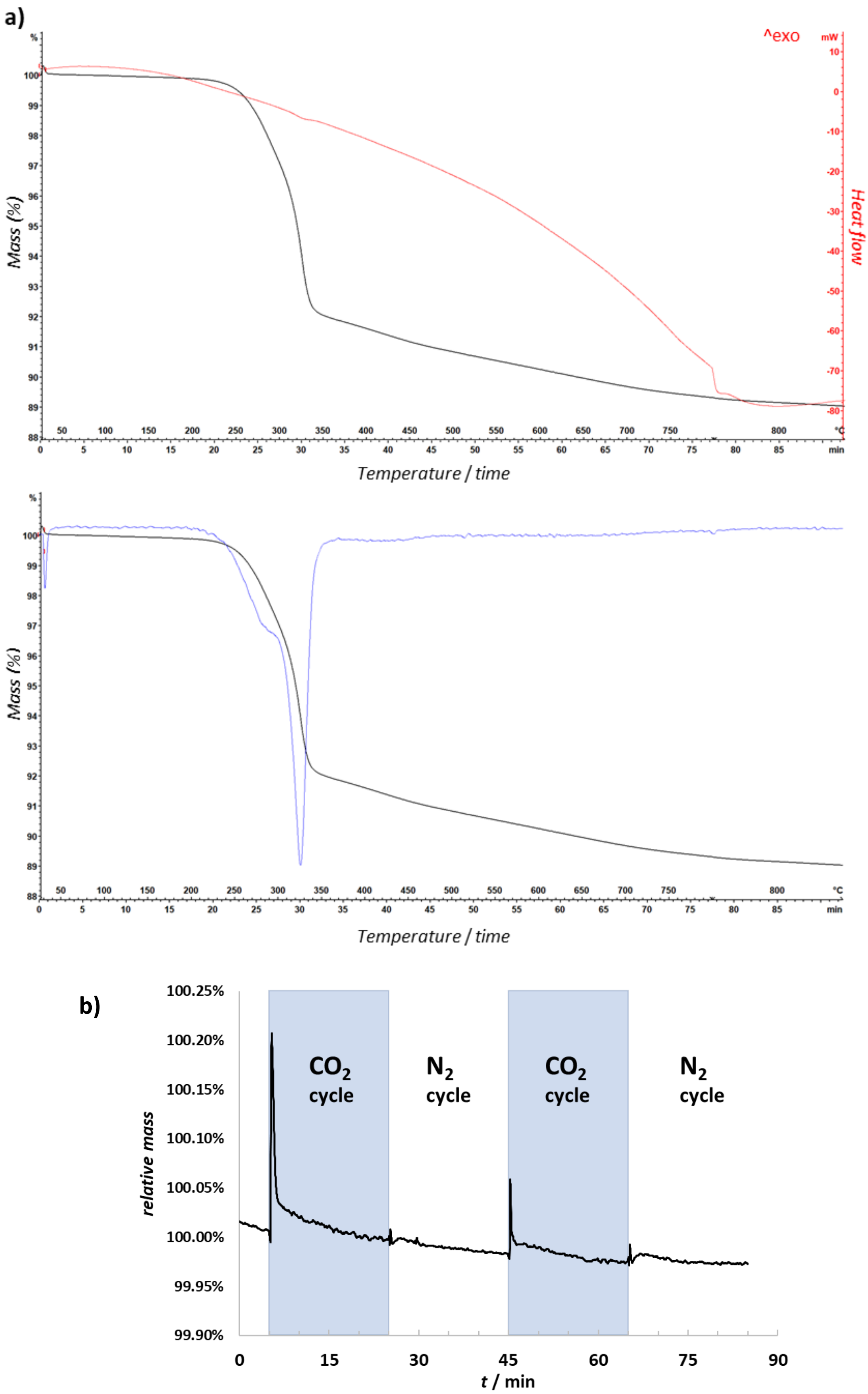

Figure S9. (a) TGA (black), DSC (red), and 1st derivative of weight (blue) curves of polymer **2** heated from 25 to 800 °C at a heating rate of 10 °C min^{-1} under a nitrogen atmosphere, and (b) thermogravimetric CO_2 adsorption curve of **2** at approximately 306 K.

5. IR spectra

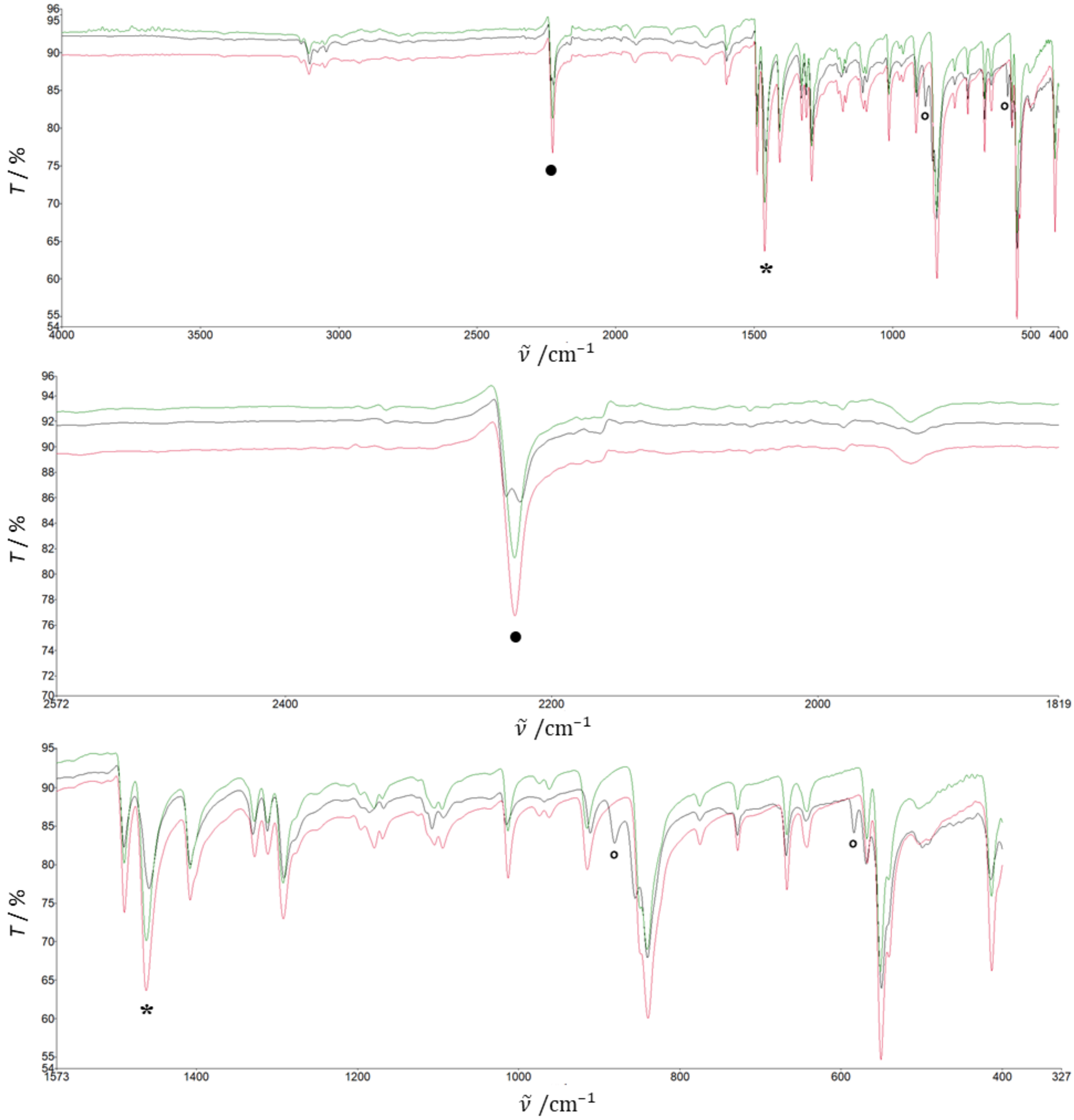

Figure S10. IR spectra of **1a** (black), **1b** heated to 190°C (green), and **1b** heated to 210 °C (red). Signals attributed to the C≡N stretching vibration band (around 2220 cm^{-1}) are marked with ●, signals attributed to the N=N stretching of the azoxy bond (around 1460 cm^{-1}) are marked with *, and signals in the fingerprint region that appear in the spectra of **1a** but not in spectra of **1b** are marked with o.

6. Powder X-ray diffraction of azoxy-linked triazine-based polymer

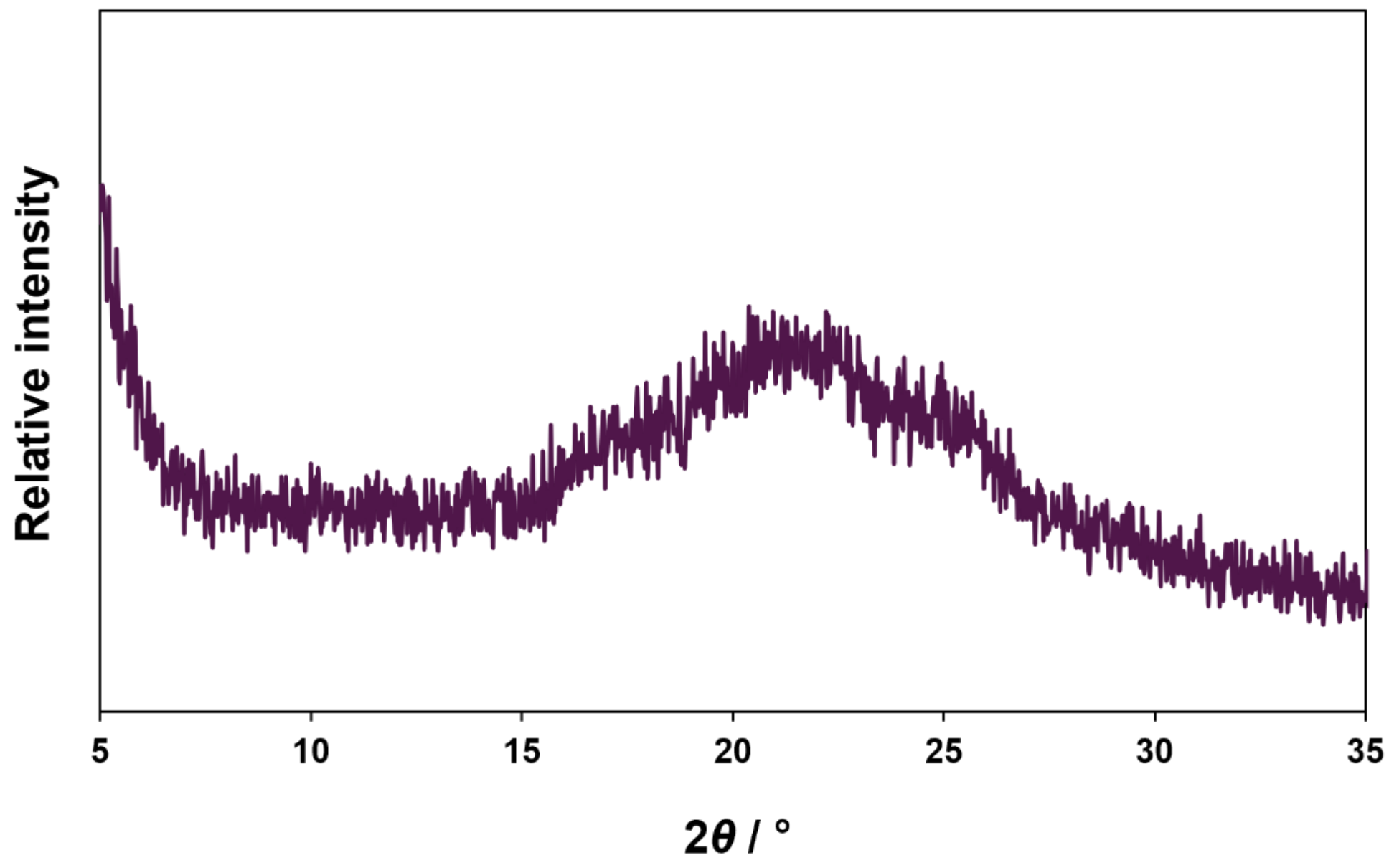

Figure S11. PXRD pattern of polymer **2**.

7. N_2 adsorption-desorption analysis

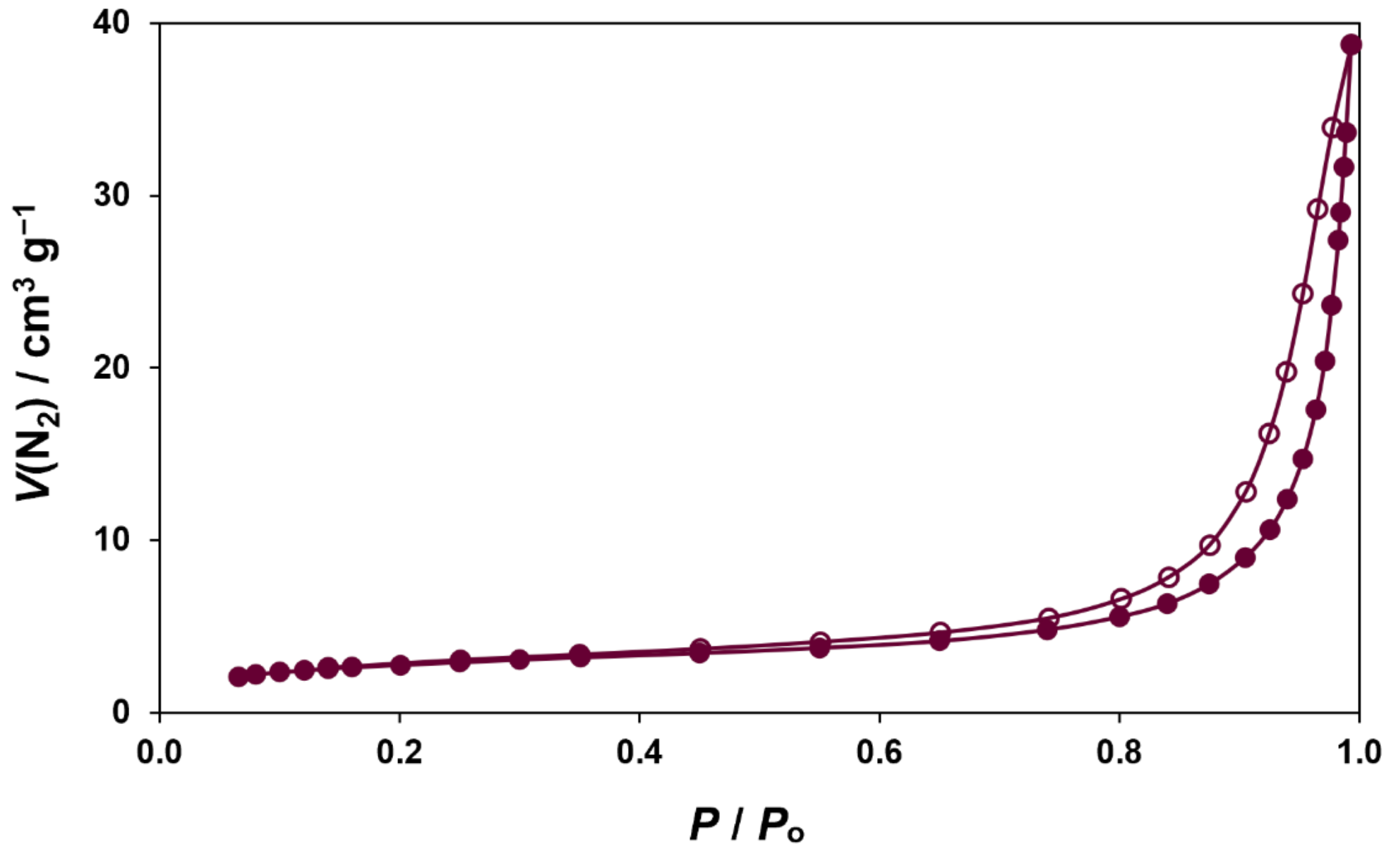

Figure S12. N_2 adsorption-desorption isotherms of polymer **2** measured at 77 K.

8. Diffuse reflectance spectroscopy

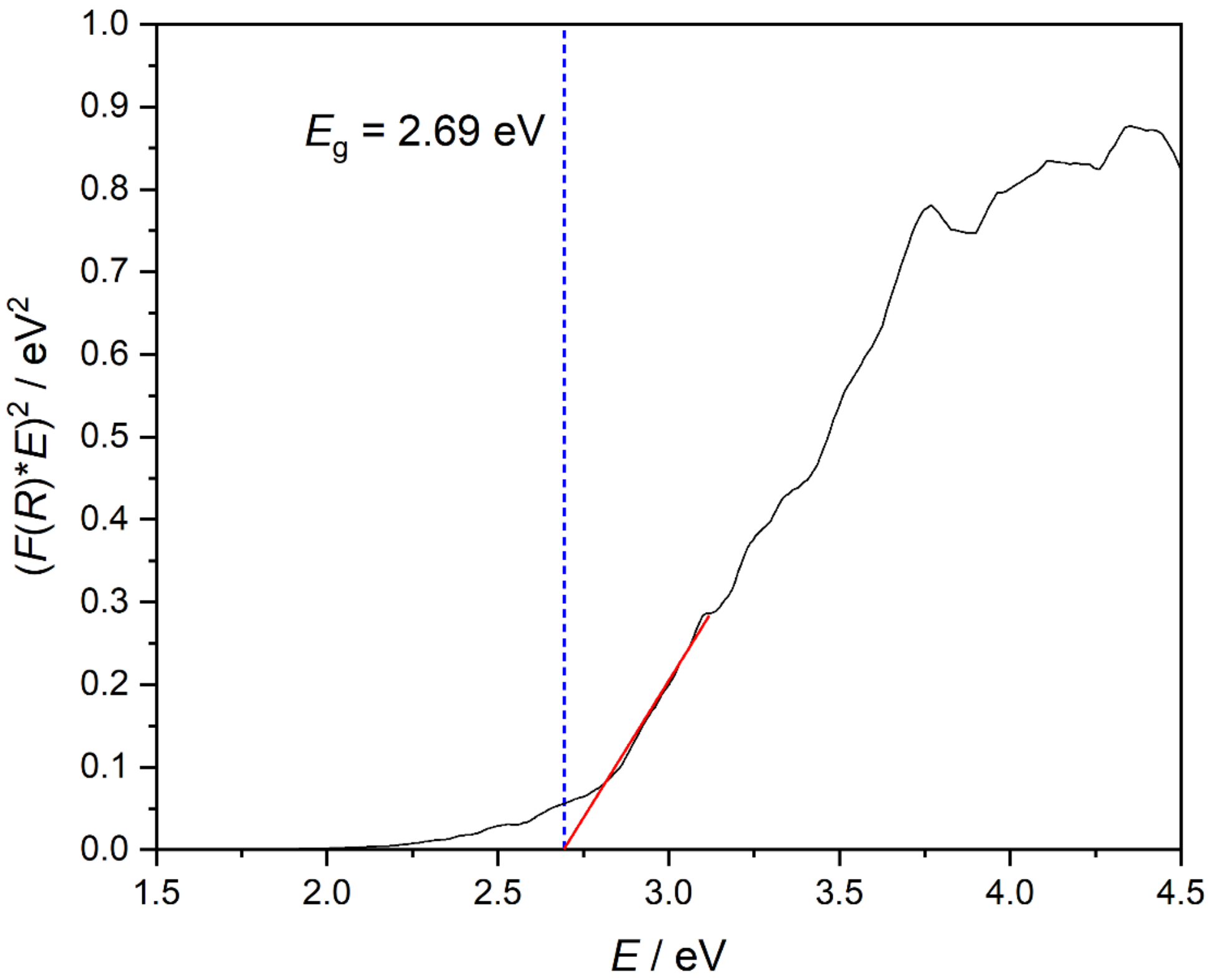

Figure S13. Kubelka-Munk plot for band gap calculation of polymer **2**.

---

[i] Cindro, N.; Car, Ž.; Petrović Peroković, V.; Borovina, M.; Panić, B.; Kodrin, I.; Biljan, I. Synthesis of Aromatic Polynitroso Compounds: Towards Functional Azodioxy-Linked Porous Polymers. *Heliyon* **2023**, *9* (11), e21781. https://doi.org/10.1016/j.heliyon.2023.e21781.

[ii] Ren, S.; Bojdys, M. J.; Dawson, R.; Laybourn, A.; Khimyak, Y. Z.; Adams, D. J.; Cooper, A. I. Porous, Fluorescent, Covalent Triazine-Based Frameworks Via Room-Temperature and Microwave-Assisted Synthesis. *Adv. Mater.* **2012**, *24* (17), 2357–2361. https://doi.org/10.1002/adma.201200751.

[iii] Guzei, I. A. An idealized molecular geometry library for refinement of poorly behaved molecular fragments with constraints. *J. Appl. Cryst*. **2014**, *47*, 806-809. https://doi.org/10.1107/S1600576714004427.
